# A systems biology framework integrating cross-species transcriptomics and PPI networks for *Xylella fastidiosa* resistance gene identification

**DOI:** 10.1186/s12870-025-07102-8

**Published:** 2025-08-11

**Authors:** Aparna S. Balan, Giorgia Tranchina, Floriana Bonanno, Tiziano Caruso, Francesco Paolo Marra, Claudio Di Vaio, Annalisa Marchese

**Affiliations:** 1 Department of Agricultural, Food and Forest Sciences (SAAF), University of Palermo, Palermo, Italy; 2 Department of Agricultural Sciences, University of Naples Federico II, Portici, Italy

**Keywords:** Resistance, Olive, Almond, Grapes, Alfalfa, RNA-Seq, Protein interaction networks, Candidate resistant genes

## Abstract

*Xylella fastidiosa*, a highly pathogenic, xylem-limited, gram-negative bacterial species, represents a significant threat to many plant species, including olive, almond, grapevine, and alfalfa. Through cross-species transcriptomic analysis of *Olea europaea*, *Prunus dulcis*, *Vitis vinifera*, and *Medicago sativa*, we identified a novel core resistance network consisting of 18 conserved genes against *Xylella fastidiosa*, alongside 1852 divergent expression patterns. These common genes may play a crucial role in orchestrating a multi-layered plant defense response, enabling (1) structural reinforcement as well as facilitating cuticular wax biosynthesis (*KCS11* and *KAS1*); (2) stress signaling mediated by hormonal crosstalk involving jasmonic acid (JA), salicylic acid (SA), and abscisic acid (ABA) mediated by the genes *AOS and CYP707A4*, alongside calcium signaling through *ACA12* gene; (3) antimicrobial 22 compound production (β-amyrin synthase *BAS*, ABC transporter *PDR6*); and (4) resource optimization through trehalose metabolism (AT1G23870) and amino acid transport (*AAP2*). The protein-protein interaction networks revealed coordinated regulation of immune hubs including BAK1, WRKY33, and WRKY40, with novel connections to subtilase proteases and ubiquitin-proteasome components. This conserved molecular framework highlights evolutionary convergence in plant defenses against xylem pathogens, providing future targets for engineering resistance through cell wall modification, stress signaling potentiation, and secondary metabolite engineering.

**Supplementary Information:**

The online version contains supplementary material available at 10.1186/s12870-025-07102-8.

## Introduction

*Xylella fastidiosa* (*Xf*) a prominent plant pathogen transmitted by xylem-sap-feeding insects, is widely known for causing significant damage to various crops on a global scale. *Xf* is a genetically diverse species that comprises three primary subspecies: *fastidiosa*, *multiplex*, and *pauca* [24]. Other subspecies such as *sandyi*, *morus*, and *tashke* have been suggested [19, 87, 89]. The ability of this pathogen to colonize different plant species and adapt to diverse environmental conditions contributes to its resilience and persistence within agricultural landscapes. Once a host gets infected by this bacterium it successfully colonizes inside the xylem vessels with its ability to alternate between motile cells and attachment-driven biofilm formation facilitates. The pathogen forms a significant bacterial load in the xylem vessels by moving efficiently within the vessels thereby increasing the likelihood of being acquired by insect vectors. For growth and survival, the bacterium acquires xylem sap, which contains water, minerals, organic acids, amino acids, alcohols, sugars, osmolytes, and vitamins, to satisfy all its nutritional requirements [52]. The blocked xylem vessels disrupt the flow of water along with the dissolved minerals from roots to leaves resulting in dehydration stress and nutrient shortage, adversely affecting the plant growth and development and leading to the death of the host plant.

The *Xf* bacterium has been reported in 40 countries across five continents, with the largest number of affected nations being in Europe, accounting for 19 of these countries. The bacterium threatens 570 host plants, seven of which are classified as major hosts, including olive, almond, grape, sweet orange, and two varieties of coffee. A total of 50 known vectors can transmit the bacterium of which 26 vectors are marked as potential (https://gd.eppo.int/taxon/XYLEFA).

The introduction of this bacterium into Europe occurred through global trade networks, resulting in the manifestation of Olive Quick Decline Syndrome (OQDS) in olive trees, first detected in 2013 in Apulia, Italy. The spread of the strain *Xf* subsp. *pauca* strain CoDiRO, ST53, in olive can occur through propagation material, but in nature, it is mainly transmitted by xylem-sap-feeding insects, such as *Philaenus spumarius* L. (spittlebug) [78, 84, 85]. The typical and most frequent symptoms are leaf scorching, with drying at the apical and/or marginal parts of the blade, drying of the canopy initially affecting isolated branches and then entire branches and/or the entire plant, and internal browning of the wood at various levels of the younger branches, branches, and trunk [38]. These symptoms are very similar to those caused by water stress. The bacterium *Xylella fastidiosa* has been included in the list of quarantine organisms of the European Union (Annex I AI of Council Directive 2000/29/EC). The infection of olive trees resulted in the death of millions of trees. The bacterium has since spread to approximately 54,000 hectares of olive trees in Apulia, raising considerable concern across the Mediterranean basin and advancing towards Central Italy [4]. The economic implications of this infection are substantial, with potential costs to Italy estimated at €5.2 billion over the next 50 years [11].

To date, no effective treatment has been identified for the control of *Xf.* A decade of research started from European Food Safety Authority and conducted by many researchers demonstrated that only the cvs. Leccino and Favolosa^®^ (FS17) exhibit a degree of tolerance to ST53 infections, characterized by milder symptoms and reduced bacterial populations in comparison to more susceptible cultivars such as ‘Cellina di Nardò’ and ‘Ogliarola salentina’ [51, 76, 90].Two subspecies of *Xf* - namely subsp. *multiplex* sequence type (ST) 6 and subsp. *pauca* - adversely affect almond production, causing almond Leaf Scorch Syndrome (ALS), leading to reduced yields and economic losses [24, 49]. *Xf* also attacks grapes, causing Pierce’s Disease with symptoms of leaf chlorosis, wilting, and diebacks in infected plants, which poses a significant threat to European viticulture especially the Mediterranean region [116]. The estimated product loss for grape production in European Mediterranean countries is approximately 0.71 billion USD [9].

The development of Next Generation Sequencing (NGS) technology has transformed genome research, allowing the sequencing of large numbers of genomes and transcripts in a short time, addressing the growing demand for deeper understanding of genetic mutations and their links to diseases [33, 37]. Nowadays, a huge amount of RNA-Seq data generated by the high throughput sequencing technique is deposited in the public repository, which can be reused to generate new knowledge and scientific findings through cross-species transcriptomics analysis approach by integrating and jointly analyzing these datasets [37, 43, 104]. The capability of cross-species transcriptomics analysis approach to combine data from multiple independent studies increases statistical power, enhances generalizability, and uncovers broader patterns or trends, which in turn pinpoints similarities, validates findings, and uncovers gene expression signatures associated with specific biological conditions, diseases, or treatments. Appropriate bioinformatic tools, techniques, and algorithms significantly enhance the cross-species transcriptomics analysis by providing ample support during the data integration, normalization, interpretation, and visualization processes. By taking advantage of modern early detection techniques and planning selection and molecular-assisted breeding programs, bioinformatics plays a crucial role in safeguarding plants [66, 86]. By sequencing the genomes of different plant varieties, researchers can identify and develop disease-resistant cultivars, with a particular focus on local genetic resources that harbour resilience genes [47]. Transcriptomics, which involves studying gene expression patterns, further enhances the understanding of plant defense mechanisms against biotic stress conditions. It also aids in the discovery of target genes that can be utilized in novel breeding techniques like genome editing [6].

Even though individual RNA-Seq studies have uncovered key findings into species-specific responses, integrating data from multiple plant species is essential for identifying both conserved and unique gene expression patterns across various host plants. Cross-species analysis also improves the robustness of findings, minimizing biases from individual studies and increasing the applicability of results. Thus, in this study, we examined RNA-Seq data from four species - *Olea europaea*, *Prunus dulcis*, *Vitis vinifera*, and *Medicago sativa -* with different levels of resistance to *Xf* and found putative genes, pathways and regulatory networks linked to resistance mechanisms.

## Materials and methods

**Fig. 1:**
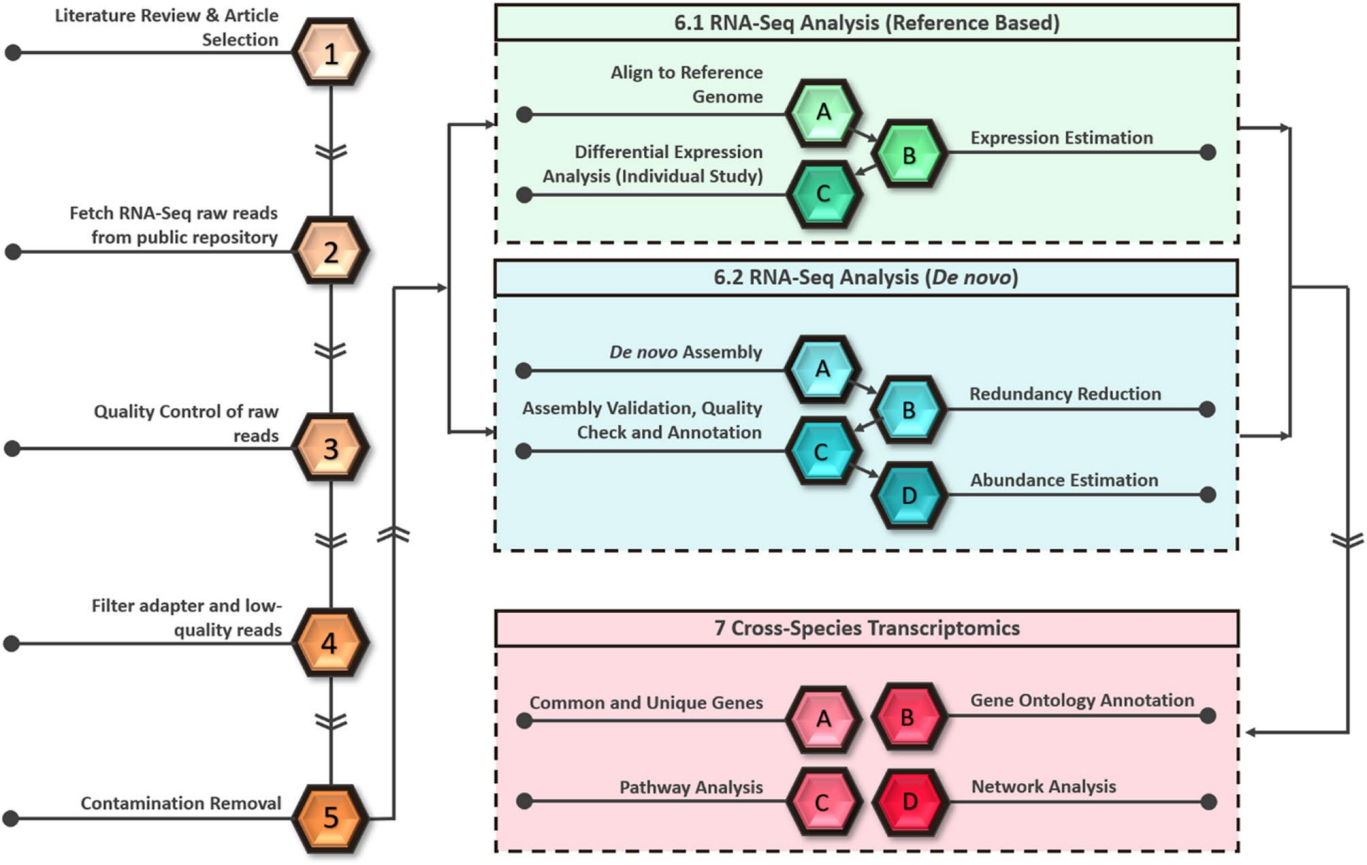
Visual representation of the cross-species transcriptomic analysis pipeline, highlighting the key stages of the workflow. The diagram outlines major components including data preprocessing, reference-based alignment and analysis, *de novo* transcriptome assembly, and the integration of results for cross-species comparison in response to *Xylella fastidiosa* infection

A visual summary of the cross-species transcriptomics analysis pipeline showing the major stages of the analysis used in this study is given in Fig. 1. Steps 1 through 5 represent the preprocessing stages of the pipeline conducted before the core RNA-Seq analysis. The pipeline is visually segmented using color codes: the green box highlights the primary steps involved in reference-based analysis, the blue box represents the stages of *de novo* analysis, and the red box features the main components of the cross-species transcriptomics analysis, including the identification of common and unique differentially expressed genes and subsequent downstream analyses.

### Data collection

An exhaustive search was conducted using PubMed and Google Scholar to identify relevant studies on *Xf* infection. This search was done using species names affected by the bacteria and included articles published up to 2023. Four studies were selected for inclusion in the analysis on the infection dynamics of *Xf* (Table 1). The selection of the studies was guided by several criteria: (1) their status as economically vital and agriculturally significant crops in regions affected by *Xf*; (2) documented predisposition to *Xf* infections based on previous research; and (3) the availability of transcriptomic resources, enabling reliable comparative analyses. These species were chosen to provide a representative framework for studying host responses to *Xf* across both woody and herbaceous plants. One of the articles examined the responses of different olive cultivars to *Xf* infection: ‘Leccino’, resistant to the disease [74] and ‘Ogliarola salentina’ highly susceptible, as reported by [31]. In addition to these contrasting olive cultivars, the selected studies explored *Xf* infection in other plant species, including *Prunus dulcis*, *Vitis vinifera*, and *Medicago sativa*. For olives, we decided to present the data for tolerant and susceptible varieties separately, leading to five comparisons (C1, C2, C3, C4, C5). For further analysis, accession IDs corresponding to the selected studies were collected. The raw sequencing data retrieval was performed using the command-line tool fasterq-dump, which is an integral component of the SRA Toolkit. The raw sequencing data associated with the identified research articles was accessed through the Short Read Archive (SRA) database, curated by the National Center for Biotechnology Information (NCBI) and served as the primary repository.

**Table 1:** Data sources for cross-species transcriptomic analysis of plant resistance to *Xylella fastidiosa* infection

Comparison	Plant	SRA ID		Read type	Article
		Control	Treated		
C1	*Olea europaea* cv. Leccino	SRR3310956 SRR3310957	SRR3310953 SRR3310954 SRR3310955	Single	Giampetruzzi et al. [31]
C2	*Olea europaea* cv. Ogliarola salentina	SRR3310961 SRR3310962	SRR3310958 SRR3310959 SRR3310960	Single	Giampetruzzi et al. [31]
C3	*Prunus dulcis*	SRR18264544 SRR18264543 SRR18264542 SRR18264541	SRR18264540 SRR18264539 SRR18264538 SRR18264537	Paired	Moll et al. [71]
C4	*Medicago sativa*	SRR10056629 SRR10056630 SRR10056631	SRR10056632 SRR10056633 SRR10056634	Paired	Abou Kubaa et al. [1]
C5	*Vitis vinifera*	SRR5851306 SRR5851303 SRR5851304 SRR5851297 SRR5851298	SRR5851301 SRR5851302 SRR5851299 SRR5851300 SRR5851305	Single	Zaini et al. [119]

### Data preprocessing, quality checking and read alignment

Raw reads from all five comparisons went through standardized processing in the same preprocessing pipeline to minimize the risk of misinterpretation due to poor data quality or contamination and to ensure consistency across species. Initially, the FastQC tool (v0.12.1) was used to evaluate sequence quality, GC content, presence of adapters, over-represented sequences, and duplication levels. Next, the Cutadapt tool (version 3.7) was applied to remove adapter sequences to ensure that only the actual biological sequences were retained for downstream analysis. For read 1 & 2, adapter trimming was performed in two steps, beginning with a 10-base minimum overlap for the first adapter, followed by the removal of the second adapter with “-b” option. Subsequently, a custom Perl script was executed to remove low-quality reads, retaining only those with a length of ≥ 30 bases, as shorter reads do not significantly contribute to the accuracy of reference genome alignment. Then, to eliminate the misinterpretation due to contamination, the bowtie2 (version 2.5.1) tool was employed for removing the contamination by filtering out reads aligning with known contaminant sequences to remove the unwanted sequences in the dataset. After eliminating rRNA contamination, the pre-processed reads were aligned to their respective reference genomes obtained from the NCBI, except for comparison C4, for which the appropriate reference genome was unavailable. The alignment was conducted using the HISAT2 tool (version 2.2.1) with the default parameters. Alignment indices were built individually for each species’ genome to ensure accurate mapping. Next, the alignment statistics were collected using the flagstat command-line tool, a component within the SAMTools (Version: 1.17) suite. As the datasets were generated using similar sequencing platforms (Illumina) and exhibited comparable quality profiles, no species-specific adjustments were made.

Because of the absence of a suitable reference genome, *de novo* transcriptome assembly procedure was applied on the pre-processed reads from C4. Trinity (v2.15.1) assembler, De Bruijn graph-based assembler, created the assembly from scratch without relying on any existing genetic blueprint. Following the initial assembly, the CD-Hit (version 4.8.1) clustering tool was applied to remove any redundant sequences among the assembled contigs to streamline the dataset and improve downstream analysis. The quality and completeness of the assembly were assessed with BUSCO (version 5.4.6) which identifies how well the assembly represents a set of conserved single-copy genes that are expected to be present in the genome. Subsequently, TransRate (version 1.0.3) was utilized for reference-free quality assessment of the assembly. Transcriptome assembly quality was further evaluated by mapping RNA-Seq reads back to the Trinity-assembled contigs using the HISAT2 aligner. The percentage of aligned reads was calculated to assess transcript completeness, complementing the BUSCO-based evaluation. The process culminates by combining the annotations for the contigs collected by blasting them against NCBI nr database, UNIPROT and TAIR database.

### Differentially expressed gene identification

The read summarization of the RNA-Seq data, for all the comparisons except C4, at gene level was performed with featureCounts (Version 2.0.6) tool. The count matrix was fed to the DESeq2, an R-language statistical pack, for the identification and analysis of differentially expressed genes using a negative binomial generalized linear model. The differential expression estimation of C4 was conducted using RSEM (v1.3.1). The expected-count obtained through the estimation was used to build feature count matrix, which is later fed to the DESeq2 package for downstream analysis. Read counts were normalized by DESeq2, which applies a median-of-ratios method that is robust to differences in sequencing depth and RNA composition. As gene expression comparisons were conducted within species (not across species), species-specific gene length biases did not impact the normalization process. Genes with an adjusted p-value less than 0.05 were regarded as statistically significant. The sequences for the differentially expressed genes were blasted against the *Arabidopsis thaliana* database to obtain the homolog TAIR IDs. The BLAST results were then categorized based on alignment score and e-value to assess the confidence of homology. “Nearly identical” matches were defined as those with a bit score ≥ 1000 and an e-value of 0, indicating exceptionally high sequence similarity. “Highly similar” matches included those with either a bit score ≥ 1000 and a non-zero e-value, or a score between 500 and 999 with an e-value of 0. These thresholds ensured that only strong and biologically meaningful alignments were prioritized for downstream analysis. Common and unique genes across datasets were subsequently identified using a custom Python script.

### Gene ontology analysis

The functional annotation of the gene and gene products were obtained with the help of Gene Ontology (GO), a robust bioinformatics resource which systematically classifies genes and their products across diverse species according to their attributes and functions. For better understanding biological roles, gene and gene products were categorized into specific functional categories such as Biological Process (BP), Cellular Component (CC) and Molecular Function (MF). Biological process involves the execution of biological programs through various molecular activities, molecular function describes specific activities performed by individual gene products, typically at the molecular level while cellular component specifies the spatial locations of gene products. The expression profiling of the common genes was carried out with the DAVID tool [93].

### Pathway analysis

The analysis and visualization of extensive genomics and transcriptomics data pertaining to individual genes was carried out using MapMan. This application was utilized to visually represent gene expression data within pre-defined metabolic pathways and functional categories using *Arabidopsis thaliana* mapping file. MapMan annotated the input data consisted of differentially expressed genes with functional categories (bins) provided by the software. These hierarchical functional categories allowed the exploration of pathways of interest.

### Protein–protein interaction network analysis

The complex interactions that underpin cellular processes was revealed through protein-protein interaction (PPI) network analysis, by mapping the physical and functional associations between proteins, resulting in the identification of key proteins that play central roles in maintaining cellular integrity or contributing to disease mechanisms. The analysis result was visualized using a graph where nodes represent proteins and edges indicate interactions. The STRING [100] database was used to build the initial network with a high confidence score > 0.9, which was later imported into Cytoscape (Version 3.10.2) software for proper layout and hub protein identification with using degree centrality as the primary metric provided by the cytoHubba plugin [16]. The cluster analysis was conducted using Molecular Complex Detection (MCODE) plug-in [3]. To find better clusters haircut option was selected with other default settings: degree cut-off at 2, node score cut-off at 0.2, k-core cutoff at 2 and maximum depth set to 100.

## Results

### Preliminary bioinformatic analysis

In this study around 1096 million raw reads were inputted for pre-processing and approximately 1090 million reads successfully passed the pre-processing stage. The aggregate range of raw reads falls approximately between 105 million to 365 million. After the pre-processing, the number of reads available for the downstream analysis were narrowed down to approximately from 104 million to 364 million (Additional File 1: Sheet 1). The pre-processed reads of *Olea europaea* cv. Leccino and cv. Ogliarola salentina were aligned to the reference genome of cv. Leccino GWHEUUU00000000.1 obtained from China National Center for Bioinformation GSA (Genome Sequence Archive) database https://ngdc.cncb.ac.cn/bioproject/browse/PRJCA015700. For *Prunus dulcis* the pre-processed reads were mapped against cv. Texas GCF_902201215.1 genome sourced from NCBI, while *Vitis vinifera* was aligned against cv. Pinot Noir 40,024 GCF_000003745.3, which was downloaded from NCBI. Due to the unavailability of an appropriate reference genome for *Medicago sativa*, all the pre-processed reads followed a *de novo* assembly pipeline. The alignment percentage for different samples of *Olea europaea* (cv. Leccino) ranges from 86.80 to 88.15%, for *Olea europaea* (cv. Ogliarola salentina) it ranges from 84.97 to 87.62%, for *Prunus dulcis* it ranges from 94.26 to 95.01%, and for *Vitis vinifera* from 91.72 to 93.35%. The total number of raw reads, the total number of reads that successfully passed the pre-processing steps, the alignment percentage for individual samples, and the average alignment percentage of each study are available in Additional File 1: Sheet 1 & 2.

For comparison C4, Trinitty assembler produced 302,564 contigs from the pre-processed reads. CD-HIT clustering tool was later applied to reduce the redundancy, and it created 239,531 non-redundant clusters with 90% similarity threshold. The assembly statistics were collected with TransRate tool and is available in Additional File 2: Sheet 1. The completeness of the assembly was assessed using BUSCO and the assessment was carried out with ‘viridiplantae_odb10’ dataset (Additional File 2: Sheet 2). The RNA-Seq reads were mapped back to the Trinity-assembled contigs to cross-verify the assembly quality (Additional File 2: Sheet 3). The annotation for the clustered contigs were gathered from NCBI nr (24454 entries) database, UNIPROT (26234 entries) and TAIR (19450 entries) database and is provided in Additional File 3. Only contigs matching ‘*Viridiplantae’* or labeled as ‘unannotated’ were retained for the downstream analysis. The count matrix generated using RSEM was used for differentially expressed gene identification.

**Table 2:** Summary of differentially expressed genes (DEGs) across five comparisons (p-value < = 0.05 and|log2(fold change)| >0.50)

Plant	Comparison	log2FC range (min-max)	Adjusted *p*-value cut-off (0.05)		
			Total DEGs	Up-regulated	Down-regulated
*O. europaea* (cv. Leccino)	C1	(−11.058304) - (08.280662)	8664	4131	4533
*O. europaea* (cv. Ogliarola salentina)	C2	(−10.166598) - (21.457315)	7136	3234	3902
*Prunus dulcis*	C3	(−03.992666) - (03.724467)	1101	550	551
*Medicago sativa*	C4	(−14.646919) - (15.473057)	10,326	4702	5624
*Vitis vinifera*	C5	(−09.312933) - (11.010041)	6582	3598	2984

### Differentially expressed genes in response to *Xf* infection

The differentially expressed genes (DEGs) were analyzed to uncover the genes whose expression levels vary between biological conditions or experimental treatments. A|log₂FC| >0.50 threshold was selected to balance sensitivity with biological interpretability in this cross-species context, where subtle yet conserved expression changes are often biologically meaningful. A stricter cutoff|log₂FC| >1 excluded many conserved genes, potentially overlooking important regulatory signals that are modest in magnitude but evolutionarily conserved and functionally relevant. The results of this sensitivity analysis are shown using an UpSet plot in Additional File 4, demonstrating that a higher cutoff would exclude many conserved expressions. This supports the decision to use a lower threshold for log2fold change. The estimation of differentially expressed genes, aimed at the identification of genes, with p-adjusted value threshold of 0.05 resulted in the detection of a total of 8664 DEGs in *Olea europaea* (cv. Leccino) among which 4131 were up-regulated and 4533 were down-regulated. In *Olea europaea* (cv. Ogliarola salentina) the comparison revealed 3234 up-regulated and 3902 down-regulated genes aggregated to 7136 DEGs. 1101 and 6582 were the total number of differently regulated genes found in *Prunus dulcis* and *Vitis vinifera*, respectively. Finally, the analysis discovered 4702 up-regulated and 5624 down-regulated genes in *Medicago sativa* (Table 2). The list of differentially expressed genes is available in Additional file 5. The normalized count for all the genes obtained during the analysis performed with DESeq2 package is available in Additional file 6_1 and in Additional file 6_2.

The variability of gene expression within a dataset is depicted in the dispersion plot (Additional file 7) generated by DESeq2, which assesses the reliability of the differential expression analysis results. In the dispersion plots of *O. europaea* cv. Leccino, cv. Ogliarola salentina and *Prunus dulcis*, most of the genes are tightly clustered around the trend line, indicating that the analysis effectively modelled the variability. In *Medicago sativa* a higher variability of genes is shown, as in *Vitis vinifera*, the genes are loosely clustered near the trendline.

MA plot in Additional file 8 with alpha threshold of 0.05 depicts the log2fold changes associated with a specific variable with respect to the average expression strength of the genes in the treatment ‘control vs treated’. The plots were generated after applying ‘apeglm’ shrinkage algorithm [122] to remove the noise generated with log2fold changes from low count genes. The differentially expressed genes are represented with blue dots, whereas grey dots represent the non-significant genes. A symmetrical distribution of points around the horizontal axis of the plot is visible in the initial four studies which indicates a lack of systematic biases whereas, in comparison five, a more scattered distribution is found.

**Fig. 2:**
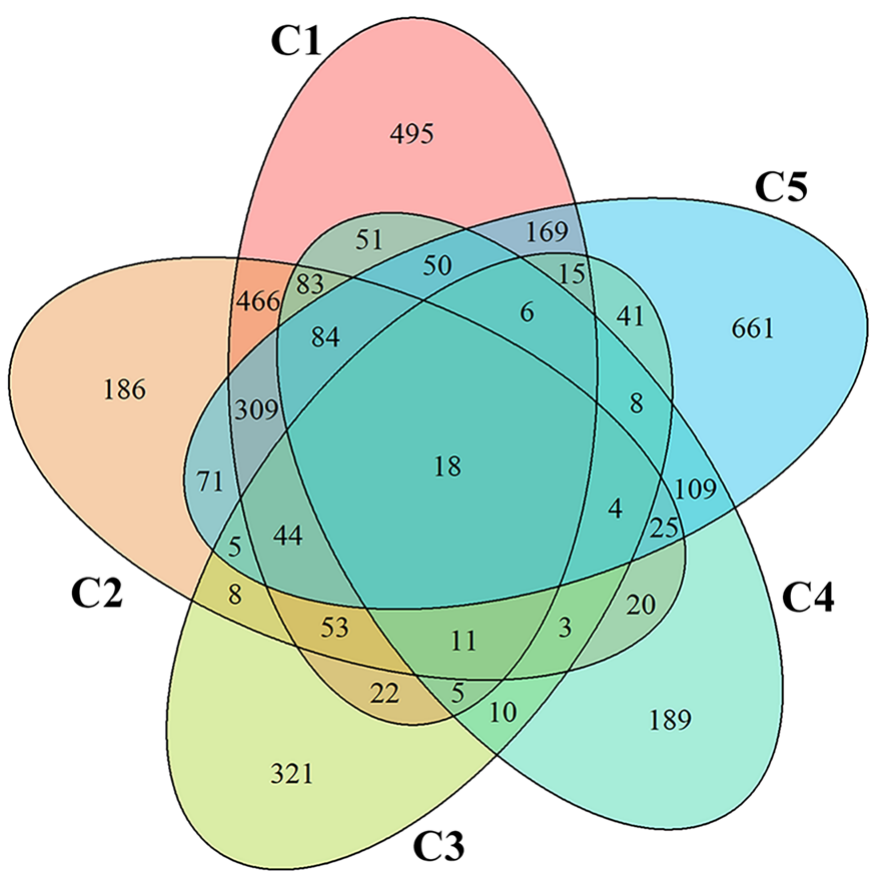
Venn diagram showing the overlapping and unique differentially expressed genes (DEGs) identified across five comparisons. The overlapping section highlights common Differentially Expressed Genes (DEGs), while the individual circles represent unique DEGs for each comparison. C1 corresponds to *Olea europaea* cv. Leccino, C2 to *Olea europaea* cv. Ogliarola Salentina, C3 to *Prunus dulcis*, C4 to *Medicago sativa*, and C5 to *Vitis vinifera*

Gene identification was performed through cross-study comparisons using BLAST alignment against the TAIR database, retaining only entries with “Highly similar” or “Nearly identical” similarity labels for TAIR ID conversion. The analysis revealed 18 common differentially expressed genes (DEGs) across all five studies, visualized in the overlapping quintuple intersection of Fig. 2’s Venn diagram and the details about these genes are available in Table 3. Species-specific unique DEGs varied substantially: *Olea europaea* cv. Leccino exhibited 495 unique DEGs, contrasted with 186 in cv. Ogliarola salentina. *Prunus dulcis* contained 321 unique DEGs, *Medicago sativa* 189, and *Vitis vinifera* showed the highest divergence with 661 unique DEGs. The genomic architecture of resistance has not been fully explored and clarified in *V. vinifera*, which could explain the significant number of unique differentially expressed genes (DEGs) identified in this species. All domesticated grapevines are susceptible to Pierce disease, suggesting that the genetic basis for resistance is lacking in *V. vinifera* [73]. This lack of resistance may lead to a higher expression of stress-related genes in response to infection, resulting in the identification of 661 unique DEGs in this cultivar. Environmental factors may also influence the expression of DEGs in *V. vinifera*, potentially exacerbating its susceptibility to *Xylella fastidiosa*. The findings imply that the unique DEGs in *V. vinifera* could be a response to the pathogen’s presence, reflecting the plant’s inability to mount an effective defense due to the absence of resistance genes found instead in its wild relatives. All common DEG expression profiles, TAIR annotations, and comparison-specific unique gene sets are systematically catalogued in Additional File 9.

**Table 3:** Common genes consistently expressed in response to *Xylella fastidiosa* across five comparative analyses

Gene name	TAIR ID	Protein name	Functional category
*TPS9*	AT1G23870	Trehalose 6-phosphate synthase	Resource allocation and transport.
*SKS5*	AT1G76160	SKU5 similar 5	Structural defense and cell wall remodelling.
*BAS*	AT1G78950	Beta-amyrin synthase	Secondary metabolism and antimicrobial effectors.
*SBT1.8*	AT2G05920	Subtilase family protein	Secondary metabolism and antimicrobial effectors.
*KCS11*	AT2G26640	3-ketoacyl-CoA synthase 11	Lipid metabolism and membrane integrity.
*PED1*	AT2G33150	Peroxisomal 3-ketoacyl-CoA thiolase 3	Lipid metabolism and membrane integrity.
*PDR6*	AT2G36380	Pleiotropic drug resistance 6	Secondary metabolism and antimicrobial effectors
*PIC30*	AT2G39210	Major facilitator superfamily protein	Structural defense and cell wall remodelling.
*OFUT20*	AT2G44500	O-fucosyltransferase family protein	Stress signaling and hormonal crosstalk.
*CYP707A4*	AT3G19270	Cytochrome P450 707A4	Stress signaling and hormonal crosstalk.
*DTX40*	AT3G21690	MATE efflux family protein	Calcium signaling and circadian regulation.
*ACA12*	AT3G63380	ATPase E1-E2 type family protein	Calcium signaling and circadian regulation.
*VEP1*	AT4G24220	VEIN PATTERNING 1	Structural defense and cell wall remodelling.
*ACA2*	AT4G37640	Calcium-transporting ATPase 2	Calcium signaling and circadian regulation.
*SDP1*	AT5G04040	SUGAR-DEPENDENT 1	Resource allocation and transport.
*AAP2*	AT5G09220	Amino acid permease 2	Resource allocation and transport.
*AOS*	AT5G42650	Allene oxide synthase	Stress signaling and hormonal crosstalk.
*KAS1*	AT5G46290	3-ketoacyl-acyl carrier protein synthase I	Lipid metabolism and membrane integrity.

### Gene ontology analysis of *Xf* associated DEGs

**Fig. 3:**
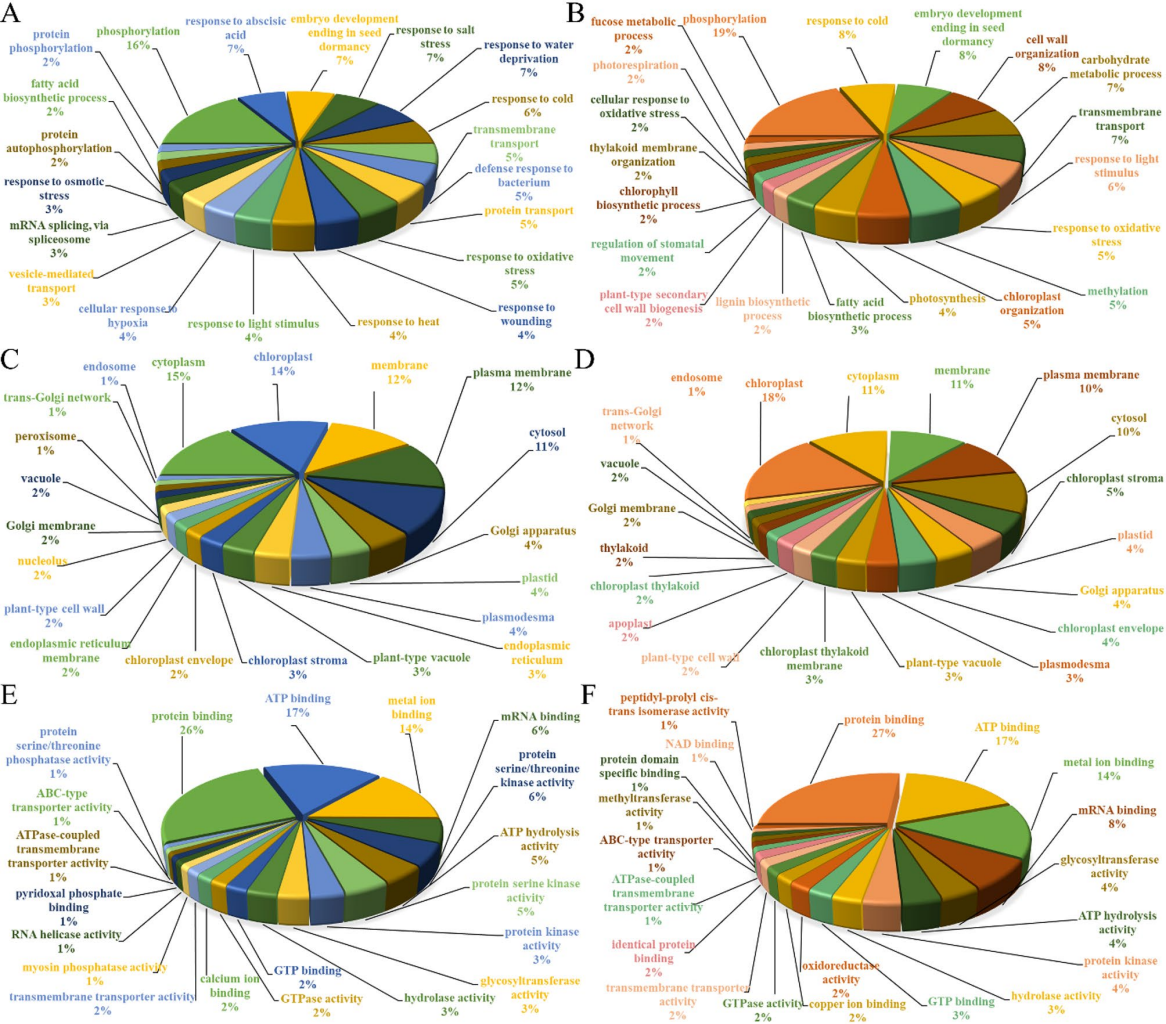
Summary of the top twenty Gene Ontology (GO) terms identified through GO enrichment analysis of differentially expressed genes (DEGs) obtained from five comparisons. Only terms with a p-value less than 0.05 are regarded as enriched. (**A**) Up-regulated BP (Biological Process) terms (**B**) Down-regulated BP terms. (**C**) Up-regulated CC (Cellular Component) terms (**D**) Down-regulated CC terms (**E**) Up-regulated MF (Molecular Function) terms (**F**) Down-regulated MF terms

Gene ontology (GO) enrichment analysis of differentially expressed genes (DEGs) identified 631 significantly up-regulated and 686 down-regulated terms (p < 0.05). Biological processes (BP) showed comparable enrichment between directions, with 345 positively and 362 negatively regulated terms (Fig. 3). Cellular component (CC) terms followed this pattern (96 up-regulated, 106 down-regulated), while molecular function (MF) terms exhibited stronger down-regulation (218 vs. 190 up-regulated). Notably, 5% of up-regulated genes mapped to ‘defense response to bacterium’ (BP). CC analysis revealed bidirectional enrichment for chloroplast, cytoplasm, and membrane localization. In MF, protein/ATP/metal ion binding dominated down-regulation, while cytoplasmic and organelle-related terms emerged in up-regulated MF annotations (Additional File 10).

### Pathway analysis

**Fig. 4:**
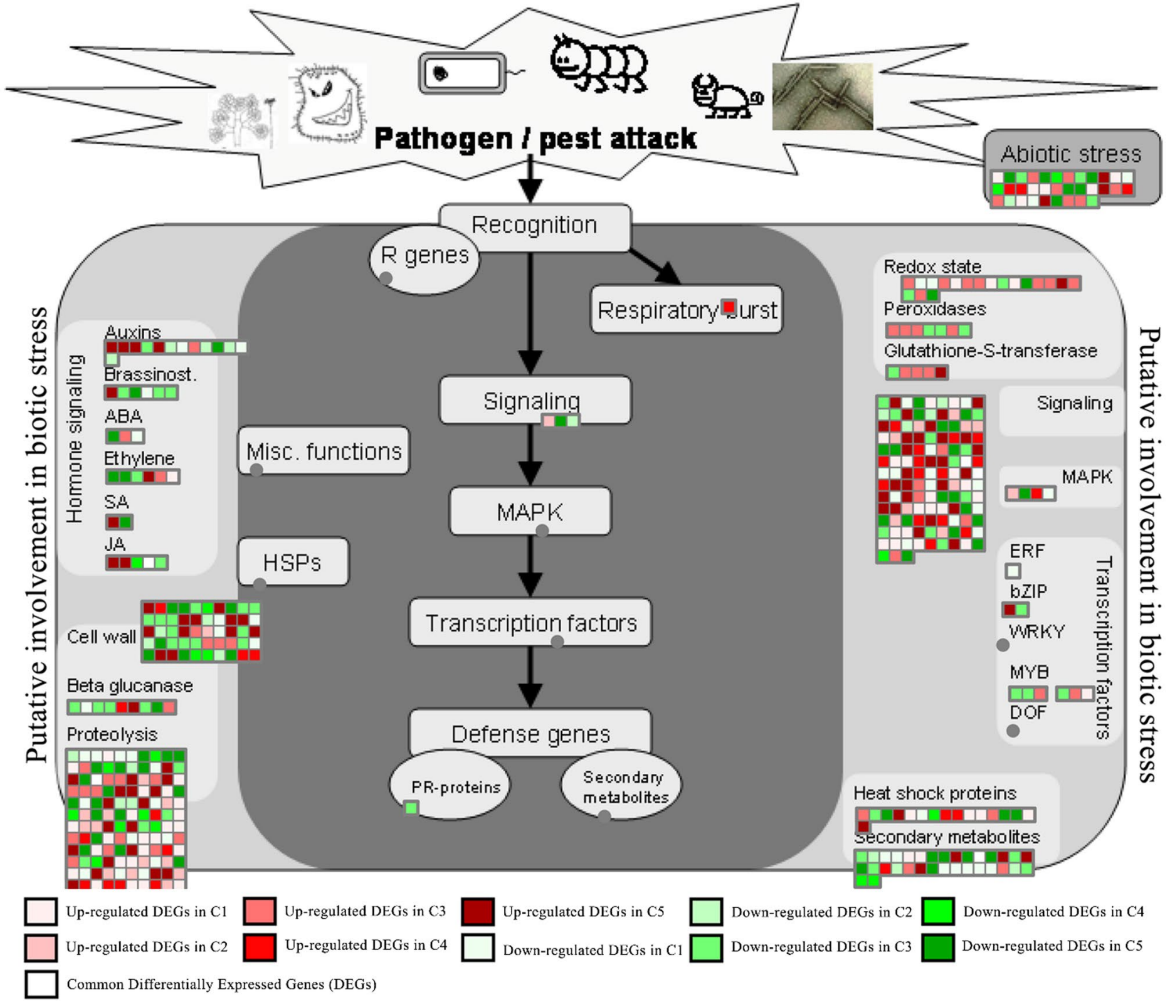
MapMan-generated general overview pathway of common and unique differentially expressed genes (DEGs) illustrating the impact of *Xylella fastidiosa* infection on the ‘Biotic Stress’ pathway, highlighting the differentially regulated genes in response to biotic stress conditions

MapMan analysis revealed substantial *Xf*-induced modulation of biotic stress pathways, with 463 differentially expressed genes (DEGs) identified in the ‘Biotic Stress’ pathway (Fig. 4). The transcriptional scenario showed 249 up-regulated genes (potentially encoding defense-related activators) and 209 down-regulated genes (indicating pathway suppression), alongside five constitutively expressed genes conserved across all comparisons - putative core regulators of biotic stress adaptation. Prominent examples include the up-regulation of the *PIN1* and *PIN6*, which enhances auxin-responsive gene expression [83, 114], and the down-regulation of the genes *TF ARF7*, which disrupt auxin transport, adversely affecting growth and defense [108]. In the brassinosteroid pathway, the gene *CYP85A2* was down-regulated [46] altering plant immunity through its interactions with other pathways [26, 53], while *BAS* emerged as a key modulator of brassinosteroid signaling, of abscisic acid (ABA) metabolism, a coordinated modulation of ABA-related signaling and growth regulatory pathways was observed where *ABA1* was down-regulated and *GCR2*, an ABA receptor, was up-regulated, potentially indicating a feedback mechanism or enhanced ABA sensitivity [22, 60]. The upregulation of *ETR2* during bacterial infection may reflect a feedback mechanism in response to elevated ethylene levels or ethylene-induced stress conditions, as ethylene receptors are often transcriptionally induced by ethylene itself [8], while the jasmonate pathway, involving *AOS* and *LOX1*, was critical for jasmonic acid production, which fortifies defense mechanisms [18, 64, 65, 109]. Moreover, the down-regulation of *ATCSLD5* shows a strategic shift in cell wall metabolism during pathogen assaults [7], and *XTH6* overexpression enhanced resistance through increased cell wall remodelling [36]. MAPK signaling networks were also implicated in plant immunity [50].

**Fig. 5:**
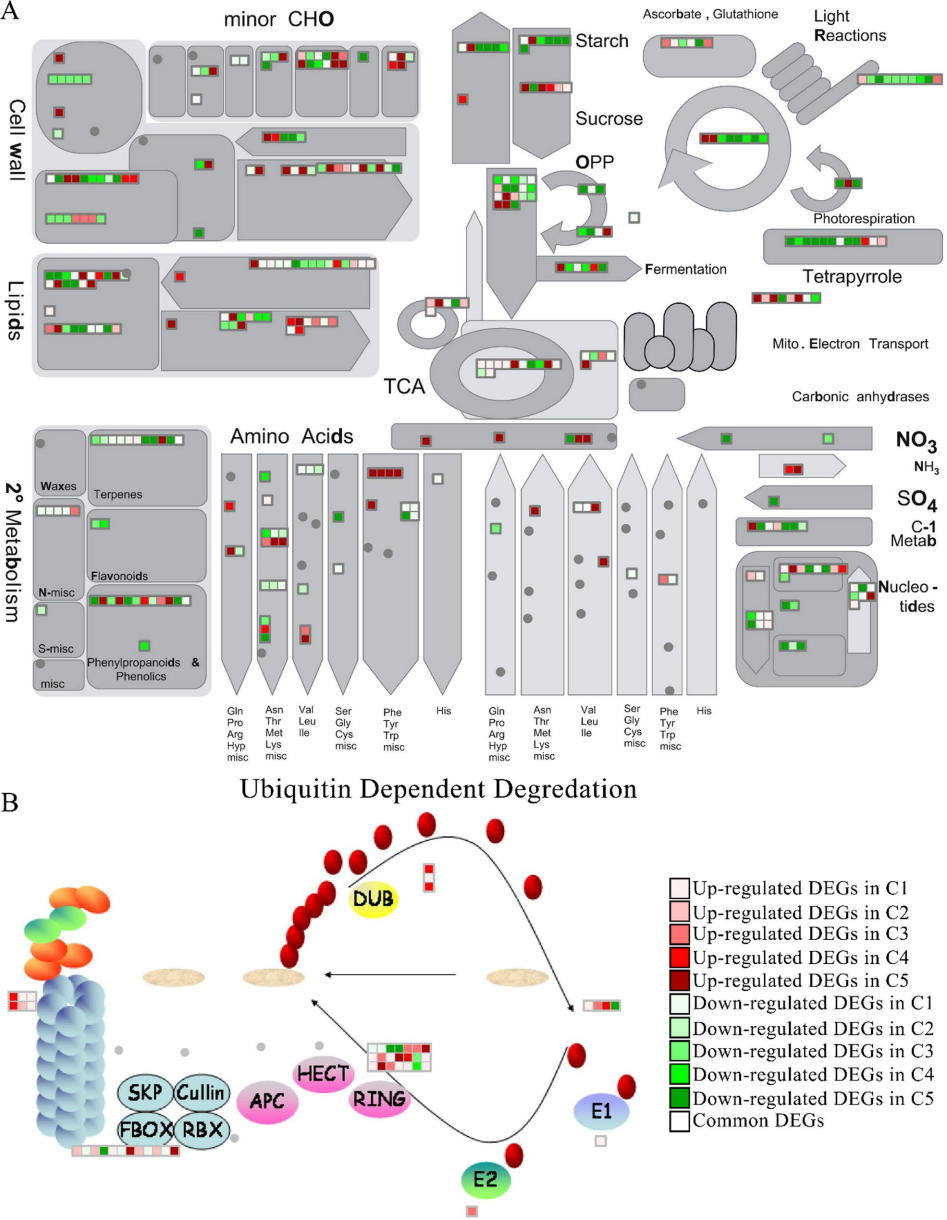
**A**) MapMan overview of metabolic activity related to shared and specific differentially expressed genes (DEGs) summarizing the overall gene expression changes in response to *Xylella fastidiosa (Xf)* infection. **B**) The proteasome pathway enriched among shared and distinct DEGs identified in response to *Xf* infection

Parallel metabolic profiling through the ‘Metabolism Overview’ pathway (Fig. 5 A) identified 376 DEGs, maintaining the five conserved genes while revealing distinct regulatory patterns: 153 significantly up-regulated versus 218 down-regulated loci. This bidirectional modulation highlights *Xf’s* dual strategy of hijacking metabolic resources while triggering counter-defense mechanisms. The list of genes along with the bin labels and descriptions are available in Additional File 11.

Proteasome profiling through the ‘Ubiquitin Dependent Degradation’ pathway (Fig. 5B) identified 48 DEGs, revealing distinct regulatory patterns: 12 significantly up-regulated versus 36 down-regulated loci. This bidirectional modulation highlights a possible *Xf*’s dual strategy of hijacking ubiquitin-proteasome system resources while triggering counter-defense mechanisms.

### PPI network construction and analysis

**Fig. 6:**
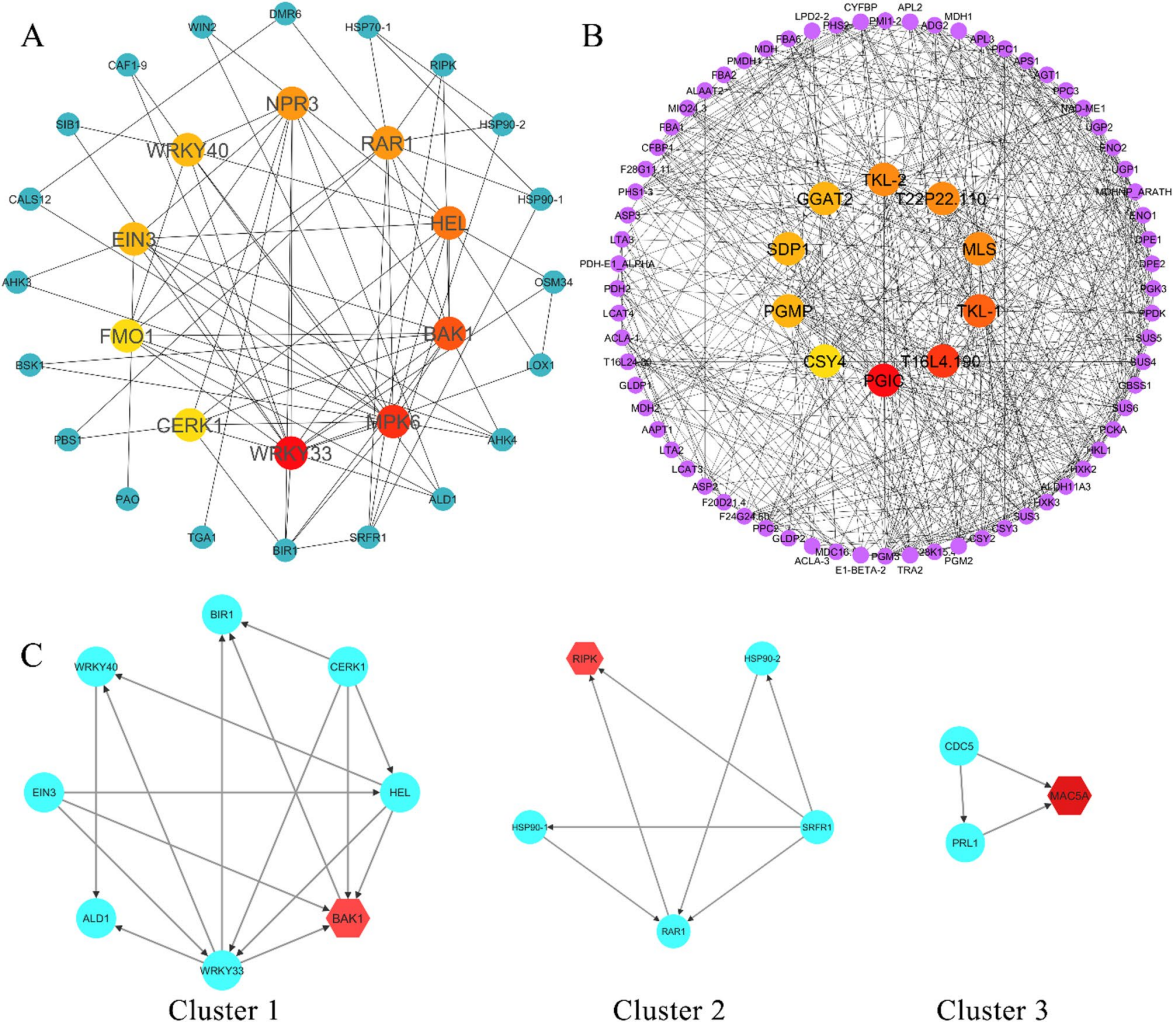
**A**) Protein-protein interaction (PPI) network of defense-related proteins, highlighting the 10 most significant hub proteins identified within the network. **B**) PPI network of common and unique differentially expressed genes (DEGs), showcasing the 10 leading hub proteins and their interactions. **C**) The three most relevant clusters derived from the defense related PPI network through network cluster analysis

Protein-protein interaction networks were reconstructed using STRING-derived associations (confidence score > 0.9) from differentially expressed genes, followed by Cytoscape visualization and topological analysis. Network complexity was reduced through two complementary strategies:

1. Hub prioritization: CytoHubba identified top 10 hub nodes per network via degree centrality (direct connection count), visualized as inner-circle core proteins versus outer-circle peripheral nodes.
2. Pathway pruning: First-neighbor interactions of hub proteins were retained to emphasize primary signaling axes.

The newly constructed network only shows the first-stage nodes displaying the shortest path. In the PPI networks, the inner circle shows the hub proteins, whereas the outer circle shows the other proteins.

The defense-specific PPI network (Fig. 6 A, confidence > 0.4) revealed 10 highly interactive proteins - WRKY33, MPK6, BAK1, HEL, NPR3, RAR1, EIN3, WRKY40, CERK1 and FMO1 - where WRKY33, MPK6, and BAK1 as the top interactors among 10 critical hubs - all established immune signaling components.

Contrastingly, the pan-DEG network (Fig. 6B, degree cutoff ≥ 15) identified PGIC (plastidial glucose transporter) and CSY4 (citrate synthase) as central metabolic hubs, alongside tandem kinases TKL-1/TKL-2 (transketolase-like proteins) and starch metabolism regulators (MLS, PGMP). This topology suggests a two-pronged adaptation to *Xf* infection:

1. Resource reallocation: Upregulated carbon metabolism (GGAT2, SDP1) and lipid remodelling enzymes.
2. Signaling rewiring: High-degree interactors T16L4.190 (uncharacterized transporter) and T22P22.110 (ABC transporter-like).

MCODE clustering revealed three functional modules, with BAK1 (LRR receptor kinase) serving as the seed node of Cluster 1 (Fig. 6 C). This defense-signaling nexus contained pathogen-responsive receptor kinases (e.g., CERK1, BAK1) and PR protein interactors (thaumatin-like proteins, β−1,3-glucanases). Full network annotations– including kinase domains (TKL-1/2; IPR017441), metabolic domains (PGIC; PF00359), and cluster-specific interactomes– are detailed in Additional File 12.

The species-specific PPI networks in Additional File 13 depict the core hub proteins in each species. These species-specific interactions may underlie the differences in how each host responds to *Xylella fastidiosa* infection, potentially influencing the severity of symptoms, effectiveness of pathogen restriction, and overall susceptibility or tolerance. Each network highlights the top 10 hub proteins. The hub proteins—including T16L4.190, T22P22.110, USP, MLS, and CSY4—are shared between comparisons C1 and C2, while unique proteins in each network highlight their differing resistance to *Xf*. The presence of SDP1 as a common protein in the species-specific networks constructed for C2 and C4 suggests a shared metabolic adaptation strategy, where both species rely on sugar metabolism to support their defense mechanisms. The presence of PGMP, a starch metabolism regulator, in both C4 and C5 indicates the commonality points to functional convergence in their response to bacterial pathogens.

## Discussion

We identified a novel, conserved core set of 18 genes shared across four phylogenetically diverse species (*Olea europaea*, *Prunus dulcis*, *Vitis vinifera*, and *Medicago sativa)*, suggesting the existence of a fundamental and ancient molecular module underpinning resistance to *Xylella fastidiosa*. These genes represent key players in stress response, signaling, and defense mechanisms essential for plant resilience. Their roles are highlighted below, drawing from their known functions in other species and in various stress conditions:

### Structural defense and cell wall remodelling (Common genes)

*SKS5* (AT1G76160), a multicopper oxidase gene is associated with the regulation of the apoplast fluid proteome during systemic acquired resistance (SAR) [12, 39]. *SKU5* has been implicated in modulating plant responses to bacterial pathogens, including *Pseudomonas syringae* [45]. Its role in enhancing pathogen resistance suggests its potential contribution to *Xf* resistance through proteome modification in the host’s extracellular space.

The gene *PIC30* (AT2G39210) is involved in the regulation of plant cell-wall composition and responses to biotrophic pathogens [94]. Its role in modulating the plant’s response to cell wall damage suggests a possible function in enhancing the plant’s resistance to *Xf* by fortifying physical barriers against pathogen invasion. *VEIN PATTERNING 1* (AT4G24220), primarily associated with vein architecture, has also been linked to defense responses [40, 102]. Its upregulation during *Xf* invasion may play a role in preserving vascular integrity.

### Stress signaling and hormonal crosstalk (Common genes)

The O-fucosyltransferase (AT2G44500) *OFUT20* or *F4I1.31* gene is associated with hormone signaling, particularly in response to pathogen infections [77, 118]. Hormones like jasmonic acid (JA) and salicylic acid (SA) are crucial for systemic defense responses [106]. Its involvement in *Xf* resistance may relate to modulation of these signaling pathways, improving the plant’s immune function. The Allene oxide synthase (*AOS*) - AT5G42650 - is involved in the biosynthesis of jasmonic acid, a hormone critical for the plant’s defense against necrotrophic pathogens [29, 97]. *AOS*’s role in enhancing jasmonate production suggests it may be integral to the plant’s ability to respond effectively to *Xf*. *CYP707A4* (AT3G19270) is a cytochrome P450 enzyme that plays a key role in the catabolism of abscisic acid (ABA), a hormone involved in stress responses. *CYP707A4* helps regulate ABA levels, which in turn influences the plant’s resistance to osmotic stress [15] and possible pathogens. Its activity may enhance *Xf* resistance by modulating the plant’s stress response pathways.

### Lipid metabolism and membrane integrity (Common genes)

The *KCS11* gene (AT2G26640) encodes a ketoacyl-CoA synthase, which is crucial in the synthesis of fatty acids and waxes, contributing to the plant’s resistance to abiotic and biotic stresses, including cold and salt stress [62]. Given that *Xf* infection triggers oxidative stress, *KCS11*’s role in maintaining cell membrane integrity and lipids could help mitigate damage and support resistance. The gene *PED1* or *KAT2* (AT2G33150) is a gene encoding a thiolase with demonstrated importance in β-oxidation and lipid metabolism [30]. This suggests a potential role in mitigating the oxidative burst triggered by *Xf* infection. The gene *KAS1* (AT5G46290) is involved in fatty acid biosynthesis, and according to TAIR, it can have a crucial role in plant defense mechanisms. The production of fatty acids during pathogen stress can influence membrane integrity and synthesis of defense molecules.

### Secondary metabolism and antimicrobial effectors (Common genes)

The *BETA-AMYRIN SYNTHASE* (*BAS* - AT1G78950) gene is crucial for the formation of triterpenoid compounds, which play an essential role in plant defense against pathogens and may be involved in SAR [39]. In this context, this gene might contribute to the synthesis of protective compounds, enhancing defense mechanisms against *Xf* in woody species like olive and grapevine. The *PDR6* or *ABCG34* (AT2G36380) is a member of the ABC transporter family and has been shown to mediate the efflux of the defense compound camalexin in *Arabidopsis* [68]. By enhancing the removal of toxins or secondary metabolites produced during *Xf* infection, *PDR6* likely supports the plant’s ability to resist infection. Plant immune signaling largely relies on post-translational modifications to establish a rapid and appropriate defense response to different pathogen types and infection pressure. [68] demonstrated that *PDR6* plays a major role in transporting camalexin, as indicated by the significantly lower camalexin secretion in *PDR6* mutants compared to wild-type plants. The phosphorylation of *PDR6* in *Arabidopsis* by two AGC kinases, OXI1 and AGC2-2, led to increased camalexin levels on leaf surfaces and decreased levels in the leaf interior, enhancing resistance to pathogens such as the Gram-negative bacterium *Pseudomonas syringae* and the fungus *Botrytis cinerea*. To date, no QTLs have been mapped specifically for resistance to *Xylella fastidiosa* in olive. However, *BAS* and *PDR6* genes were identified in Giampetruzzi (2016) ’s RNA-seq study, and the same genes were also detected in *Leccino* progenies by [51]. Notably, in La Notte’s study (2024), resistant seedlings S215 and S234 appeared to escape infection 24 months post-inoculation, with most of their progenies—especially those of S215—showing recovery. *BAS* and *RD6* were among the DEGs common to both genotypes. *SUBTILASE 1.8* (AT2G05920) genes are serine proteases that have been shown to regulate plant immune responses by modulating the activity of other defense-related proteins [39]. Subtilase genes could be involved in processing proteins that activate immune pathways in response to *Xf* infection.

### Resource allocation and transport (Common genes)

The *TREHALOSE-6-PHOSPHATASE SYNTHASE 9* (AT1G23870) gene is involved in the biosynthesis of trehalose, a sugar known for its protective role under stress conditions [88]. In *Arabidopsis*, trehalose accumulation has been linked to enhanced tolerance against osmotic stress, bacterial infections, and heat stress (reviewed by [21]. Given the environmental stressors associated with *Xf* infection, AT1G23870 may help in maintaining cellular integrity during pathogen-induced stress. The gene *SDP1* (AT5G04040) is involved in sugar metabolism, which is essential for energy production during stress responses. In the case of *Xf* infection, *SDP1* could support the energy requirements needed for defensive responses, such as the synthesis of secondary metabolites and reactive oxygen species (ROS). The *AAP2* (AT5G09220) is an amino acid permease that regulates amino acid transport, which is critical for plant immune responses [14]. During *Xf* infection, *AAP2* may help facilitate the uptake of essential amino acids needed for the synthesis of defense-related proteins and metabolites.

### Calcium signaling and circadian regulation (Common genes)

*ACA2* (AT4G37640) and *ACA12* (AT3G63380) are calcium-transporting ATPases that play distinct roles in calcium signaling during plant immune responses, differing in their localization, regulatory mechanisms, and functions. *ACA12* is situated at the plasma membrane and operates independently of calmodulin, enabling rapid calcium efflux that supports early immune responses such as pathogen-triggered immunity (PTI) and stomatal closure, which are essential for blocking pathogen entry. In contrast, *ACA2* which is localized to the endoplasmic reticulum, where it is not directly activated upon pathogen perception, maintains the internal Ca²⁺ balance required for proper functioning of downstream signaling pathways, including those controlling gene expression and cell survival [59, 79, 117]. The *MATE efflux family protein* (AT3G21690) gene is involved in aluminium stress [95] and circadian rhythm regulation, which has been shown to influence plant immune responses. Plants with well-regulated circadian rhythms are more effective in defense responses against pathogens [42]. Its role in *Xf* resistance may be connected to the timing of immune activation.

The conserved genes illustrate a comprehensive strategy against *Xf*, consisting of several layers:

First, pre-invasive defenses such as cuticular waxes (*KCS11*, *KAS1*) and possible cell wall reinforcement (*PIC30*) to block pathogen entry. Next, stress signaling mechanisms, including hormonal crosstalk (*AOS*, *CYP707A4*) and calcium signaling (*ACA12*), amplify immune responses. Following invasion, post-invasive responses deploy antimicrobial compounds (*BAS*, *PDR6*) and proteolytic activation (*SUBTILASE 1.8*) to target *Xf* proliferation. Finally, resource optimization through trehalose (AT1G23870) and amino acid transport (*AAP2*) ensures metabolic demands are met during infection.

### Biotic stress pathway

The biotic stress pathway elucidates how plants regulate defenses against *Xf* biotic stressors through hormone interactions. Salicylic acid induces calcium ion (Ca²⁺) bursts by activating cytosolic Ca²⁺ channels, which subsequently amplify immune responses through the activation of pathogenesis-related (PR) genes. However, *Xf* can subvert this process by degrading SA or inhibiting PR gene expression. Jasmonic acid promotes anti-herbivore responses, thereby prioritizing these defenses over SA-mediated immunity. It activates *ACA2*, a plasma membrane Ca²⁺-ATPase, which exports cytosolic Ca²⁺, thereby diminishing the synergy between SA and calcium signaling. This antagonism may be exploited by *Xf* to weaken plant defenses further, as the regulation of calcium homeostasis is crucial during stress responses [28, 34, 75]. Abscisic acid activates *ACA12* under drought stress, sequestering Ca²⁺ into the endoplasmic reticulum and reducing cytosolic Ca²⁺ levels. *Xf* infection is perceived as a condition of drought stress, elevating ABA levels. This can consequently impact calcium signaling pathways that are vital for plant immunity [13]. In the early stages of infection, SA predominates, while in chronic stages, ABA and JA become dominant, leading to suppression of defense mechanisms. This shift in hormonal balance reflects the complex regulatory networks that govern plant responses to pathogens, highlighting the need for further exploration of these interactions [55, 119]. For instance, it would be crucial to check how hormonal levels change and modulate during the infection. Auxins play their part by balancing growth and defense through *PIN1* and *PIN6* genes [83, 114]. Brassinosteroids enhance defenses by activating stress-related genes [46], while ethylene and jasmonate pathways synergize to combat *Xf* (AOS/LOX1; [18, 65]. Cell wall integrity is affected by *ATCSLD5* gene [7] but reinforced by *XTH6* gene [36]. Immune regulation involves *MLO* genes, MAPK signaling [50], and the ubiquitin-proteasome system [121], with *BAS* further enhancing immunity through brassinosteroid signaling crosstalk [26]. These insights into molecular mechanisms provide valuable targets for improving crop resilience and promoting sustainable agriculture.

### Metabolic overview

MapMan’s metabolic overview offers a visual representation of how bacterial infections influence plant metabolism, showing the significant alterations in metabolic pathways that occur upon infection. The plant cuticle is a lipid-based polymer layer coated with waxes that covers the outermost surfaces of plant organs, acting as the first line of structural defense against pathogens. Instead of remaining static, the cuticle undergoes dynamic remodelling during pathogen invasion and is involved in plant immune signaling. The common genes involved in various metabolic pathways include *KAS* and *KCS* in lipid metabolism, *ATTPS9* in carbohydrate metabolism, *PKT3* in amino acid metabolism, and *BAS* in secondary metabolism.

The β-Ketoacyl-ACP Synthase (*KAS*) genes are integral to the fatty acid biosynthesis pathway in plants [58], producing fatty acids essential for constructing lipid-based structures like cell membranes and the cuticular layer. These structures serve as primary physical barriers against pathogenic invasions, including bacterial infections. Fatty acids synthesized through the KAS pathway are precursors for signaling molecules such as jasmonic acid (JA), which play pivotal roles in activating plant defense mechanisms. The *KCS* gene family plays a key role in producing very-long-chain fatty acids (VLCFAs), essential for forming protective plant barriers like cuticular waxes and suberin. These barriers help resist pathogens. Research shows some *KCS* genes are activated during infection while others are suppressed, indicating a gene-specific regulatory system that shapes plant defense responses (e.g. [82].

The *ATTPS9* gene, classified under the minor CHO (carbohydrate) section, encodes an enzyme involved in trehalose-6-phosphate synthase activity, which is crucial for stress responses and energy regulation in plants [107]. The *PKT3* gene, associated with amino acid metabolism, encodes an enzyme involved in polyketide biosynthesis—secondary metabolite that influence plant-pathogen interactions—while polyketide synthase (PKS) pathways, including the PKS10-2 pathway, are crucial for the pathogenicity of certain plant pathogens and may play roles in defense mechanisms against bacterial infections in host plants (e.g. [103]. The β-Amyrin Synthase (*BAS*) contributes significantly to the plant secondary metabolism by catalyzing the production of β-amyrin, which can induce the production of ROS, suggesting its potential as an antimicrobial agent [39].

### Ubiquitin dependent degradation

In plants, protein degradation is tightly regulated by the ubiquitin–proteasome system (UPS), which serves as a core component of immune and stress responses. In addition to its proteolytic role, the UPS—through its 20 S RNase activity—may participate in a still unidentified antiviral defense mechanism by modifying the proteome to enhance survival. Variations in ubiquitin levels, as well as in the activity of E1 and E2 enzymes, can lead to widespread cellular reprogramming during defense, while E3 ubiquitin ligases, which determine substrate specificity, are critical players in plant–pathogen interactions [20]. The up-regulation of *UBQ4*, a polyubiquitin gene, during pathogen infection indicates an increased demand for ubiquitin monomers to support the ubiquitin–proteasome system (UPS), which is essential for tagging and degrading misfolded or regulatory proteins, allowing the plant to fine-tune its immune response [99]. The expression of the E1-activating enzyme gene *SAE2* and the SUMO‑conjugating E2 enzyme *UBC9* enhances post-translational modifications critical for immunity [10]. Zinc finger E3 ligases facilitate selective protein ubiquitination, modulating immune receptor levels, signaling intermediates, and transcriptional regulators essential for both PTI and ETI pathways [115]. F-box proteins dynamically regulate target recognition for SCF complexes, impacting defense signaling and strengthens immunity [98], whereas degradation of key F-box proteins like *COI1* [35] hinders jasmonate signaling and compromises immune responses. The marked induction of proteasome subunit expression shows that, during basal defense, the plant boosts proteasome activity to rapidly degrade specific proteins, helping to regulate and fine-tune immune signaling, remove harmful or unnecessary proteins, and thereby strengthen its ability to resist infection [105]. However, to date, there is no evidence that *Xylella fastidiosa* interferes with proteasome activity to disrupt phytohormone signaling; therefore, further studies should investigate whether *Xf* effectors can interact with the UPS. This could be achieved through effector screening approaches, such as yeast two-hybrid assays or affinity purification–mass spectrometry, to identify potential interactions between *Xf* secreted proteins and host UPS components.

### Hub proteins

The protein-protein interaction (PPI) analysis revealed a complex network of genes involved in the response to *Xylella fastidiosa* infection. By mapping these interactions several highly interconnected hub nodes emerged which suggested the presence of functional pathways and modules related to resistance. In particular, the analysis of interactions between defense-related proteins highlighted several proteins known for their involvement in immune responses, such as BAK1 (cluster 1 Fig. 6 A, C), RIPK (cluster 2, Fig. 6 C) and MAKSA (cluster 3 Fig. 6 C). The network-driven identification of BAK1 as a dual topological and functional hub, alongside metabolic regulators (PGIC, CSY4), reveals a sophisticated interplay between resource management and immune prioritization in *Xf*-infected plants. These findings align with the emerging paradigm of “defense priming through metabolic reconfiguration”, where plants optimize resource allocation to sustain energetically costly immune responses. The prominence of receptor kinases (BAK1, CERK1) and PR protein interactors in Cluster 1 underscores the activation of canonical pathogen recognition mechanisms. Its interactions with MPK6, WRKY33 and WRKY40 suggest BAK1’s central role in defense signaling [112]. WRKY33 and WRKY40 are significant in modulating immune responses, with WRKY33 linked to oxidative stress and jasmonic acid signaling, while WRKY40 enhances resistance against pathogens and abiotic stress [113].

The hub protein BAK1, also known as SERK3, plays a crucial role in plant immunity, including potential resistance to *Xanthomonas fastidiosa* and *Xylella*. One key characteristic of BAK1 is its function as a coreceptor for multiple receptor-like kinases (RLKs), which are essential for recognizing pathogen-associated molecular patterns (PAMPs) and initiating immune responses. BAK1 interacts with pattern recognition receptors (PRRs) such as FLAGELLIN-SENSING2 (FLS2) and ELONGATION FACTOR-TU RECEPTOR (EFR), facilitating the activation of immune signaling pathways upon pathogen detection [17]. Another important feature of BAK1 is its proteolytic processing, which is regulated by calcium and is critical for its function in plant immunity. The cleavage of BAK1 enhances its ability to mediate immune responses, as evidenced by the identification of the aspartic acid residue at position 287 (D287) as essential for this process. Mutations at this site, such as BAK1D287A, impair the protein’s ability to activate downstream signaling pathways, leading to compromised immune responses. BAK1’s localization to the plasma membrane is vital for its role in immunity. Proper localization allows BAK1 to interact with other RLKs and initiate signaling cascades that lead to defense responses against pathogens like *Xf*. The D287 mutation not only affects BAK1’s cleavage but also reduces its plasma membrane localization, further highlighting the importance of this residue in maintaining BAK1’s functional integrity in plant defense against *Xanthomonas* and *Xylella* in transgenic sweet orange and *Arabidopsis* [32, 69]. Notably, [51] also reported *BAK1* among the DEGs in the progenies of resistant genotypes S234 and S215, further supporting its potential role as a hub gene in the defense response against *Xylella fastidiosa*.

MPK6, a mitogen-activated protein kinase, integrates defense signals and activates downstream responses, including ROS production [111]. Proteins like NPR3 and EIN3 are part of hormonal signaling networks that mediate defense responses, with NPR3 regulating SAR [25] and EIN3 involved in ethylene signaling against pathogens [120]. CERK1, known for chitin perception, may indicate broader defense mechanisms against *Xf*. ALD1 is another interesting node identified in the network, having a relationship with BAK1. In model plants, it has been demonstrated that ALD1 plays a crucial role in establishing robust defense responses against pathogens. The ALD1 mutant is shown to be hypersusceptible to the virulent bacterial pathogen *Pseudomonas syringae*, which correlates with a reduced accumulation of the defense signal salicylic acid (SA). This suggests that ALD1 is vital for effective immune responses, particularly in the context of pathogen resistance [96].

Another important node is represented by HEL (Hevein-like) genes, crucial in plant defense responses to biotic stress, particularly through their interaction with various hormones [2]. Reactive electrophile species, which accumulate during pathogenesis, can activate HEL gene expression, indicating their role in the plant immune response. Specifically, the expression of HEL is upregulated by exogenous OPDA (oxo-phytodienoic acid), a compound that is part of the jasmonate family, highlighting the connection between HEL genes and jasmonate signaling [2]. Moreover, the study suggests that HEL gene expression is also influenced by salicylic acid and other low-molecular-mass regulators, which are known to control defense gene expression in plants. This interplay between HEL genes and signaling molecules like jasmonate and salicylic acid underscores the complexity of the plant defense mechanisms against biotic stress [2].

The initial events associated with pathogen perception and responses to environmental stresses encompass the generation of ROS (e.g. [70] and nitric oxide (review in [92], both of which are integral to the disease process, although their precise roles remain the subject of ongoing investigation. Along with ROS and reactive nitrogen species, reactive electrophile species (RES) play key role in regulating defense genes. They may also cause damage to both host and pathogen cells, which is a common aspect of the plant’s response to non-virulent pathogens [2].

Since it is well known that *Xylella fastidiosa* disrupts phytohormone networks to promote infection [5, 81], future investigations should explore whether *Xf* can interfere, for example, with HEL expression by modulating RES and ROS, potentially blocking the production of hevein-like proteins. This hypothesis is supported by the findings that suggest *Xf* employs various extracellular proteins to manage oxidative stress and facilitate biofilm formation, which is crucial for its pathogenicity [67].

In Cluster 2, Fig. 6 C, the protein RIPK is crucial for the production of ROS, which are key signaling molecules in plant defense mechanisms. These mechanisms operate across various layers of the plant immune system, including effector-triggered immunity (ETI), pathogen-associated molecular pattern-triggered immunity (PTI), damage-associated molecular pattern-triggered immunity (DTI) and SAR. Model plants with mutations in the RIPK gene produce lower levels of ROS when exposed to immune elicitors, indicating that RIPK is essential for ROS generation and consequently, for effective plant defense [56]. HSP90.1 and HSP90.2 are closely related members of the HSP90 (Heat Shock Protein 90) family in plants. As a molecular chaperone, HSP90-1 ensures the proper folding, stability and activation of various client proteins involved in defense mechanisms. It interacts with RAR1 and SGT1 to regulate the stability and function of Resistance (R) proteins, which are crucial for triggering defense signaling pathways [101]. HSP90.2 acts as a partial suppressor of the autoimmune mutant chs3-2D in *Arabidopsis thaliana* and thus it is essential for CHS3-mediated defense signaling [61]. SRFR1 plays a key role in modulating the balance between defense activation and suppression by acting as a negative regulator of effector-triggered immunity against the bacterial pathogen and thereby fine-tuning the immune system to balance defense and growth [57].

In Cluster 3, MAC5A plays a role in mRNA splicing during pathogen defense, emphasizing its relevance in gene expression regulation [72, 110].

The observed metabolic alterations among the four species, marked by the upregulation of genes associated with carbon metabolism (e.g., GGAT2, SDP1) and the regulation of ABC transporters (T22P22.110), suggest two plausible compensatory strategies employed by the plant. The first, nutrient deprivation defense, involves redirecting photoassimilates from the xylem to parenchyma cells via PGIC, to starve the vascular pathogen. The second strategy, toxin compartmentalization, suggests that ABC transporters may play a role in sequestering *Xf*-derived toxins. This has also been reported in *Citrus* species [23], where intriguingly, *Xf* in vitro counteracts the plant defense responses through osmoregulated periplasmic glucans (OPGs) synthesized by *MDOH* (XF1623) and *MDOG* (XF2682)2. These OPGs stabilize membrane integrity under nutrient stress—a direct adaptation to survive the host’s xylem-specific starvation tactics, thriving under stress conditions within the xylem of its host plants and inducing acid tolerance [23]. Such co-evolutionary dynamics are further exemplified by the redox rewiring response, which is also observed in the four species making it possible to counteract the ROS-neutralizing catalases produced by *Xf*, as discussed by [31] and [51] further illustrating the intricate interplay between the pathogen and host defense mechanisms.

The frequent appearance of BAK1 across different species’ networks suggests that these plants may depend on BAK1-driven PTI as a core component of their defense system. Its interaction with BIR1—a key suppressor of cell death and immune responses—further highlights the existence of a tightly regulated mechanism that prevents unwarranted immune activation [63]. The overlap of BAK1 and BIR1 suggests that despite evolutionary divergence, these species may maintain similar defense signaling modules, possibly indicating shared resistance strategies to biotic stressors. Similarly, the presence of RIPK (RPM1-Induced Protein Kinase) across majority of species-specific PPI networks suggests it’s functions as a conserved immune regulator, particularly through ROS-mediated defense signaling [56]. RIPK activates the NADPH oxidase RBOHD, promoting the production of ROS during pathogen attack, which is a key early event in PTI [41]. Its dual role in initiating defense responses while restraining excessive immune activation indicates a finely balanced regulatory mechanism conserved across plant species.

Defense pathways, such as cuticular wax biosynthesis, are conserved across distantly related plant species, shaped by evolutionary pressures from pathogens like *Xylella fastidiosa* and other xylem-invading microbes. The cuticle acts as a vital barrier, limiting pathogen entry and influencing susceptibility through its composition. Over time, selective pressure from such pathogens has driven the evolution of traits that strengthen plant defenses, including the development of receptor-like kinases (RLKs) that help detect pathogens and regulate protective responses like wax biosynthesis. The widespread conservation of these mechanisms highlights their critical role in enhancing plant survival and reproductive success under pathogen stress [48, 123].

*Xylella fastidiosa* counters plant resistance network through strategies such as biofilm formation and the production of effectors like oligosaccharides (OPGs). The biofilm, composed of extracellular polymeric substances (EPS), enables *Xf* to colonize xylem vessels while evading immune detection. This structure also protects the bacteria from environmental stress and interferes with the plant’s ability to mount an effective immune response [81]. In addition to forming biofilms, *Xf* secretes a range of effectors that disrupt host plant immune responses. OPGs, for example, interfere with both microbe-associated molecular pattern (MAMP)-triggered immunity (MTI) and ETI, weakening the plant’s ability to detect and respond to infection. By suppressing these defense pathways, *Xf* facilitates its colonization and systemic spread within the host [91]. The density of the bacterium populations critically influences its pathogenic success: at low cell densities, the bacterium adopts a colonization mode to infiltrate the plant, while at high densities it switches to a transmissible form that favours insect vector acquisition—this strategic shift maximizes infection spread while avoiding severe disease symptoms that might deter insect vectors. This dynamic regulation is orchestrated by quorum sensing via diffusible signal factor (DSF), which controls adhesin expression and biofilm formation—key to the switch between plant colonization and insect uptake. Notably, the interplay of biofilm maturation and effector molecules like OPGs represents a sophisticated strategy enabling *Xf* to suppress host defenses, maintain stealthy persistence, and ensure efficient propagation across both plant and insect hosts [44].

### Limitations and future scope

Although the cross-species analysis reveals conserved transcriptional patterns that may underlie common responses to *Xylella fastidiosa* infection, several factors call for discussion due to methodological constraints inherent to this approach. Variability in RNA-Seq data quality—ranging from sequencing depth to library preparation protocols—can further complicate interpretation by introducing dataset-specific artifacts. The choice of reference genome exerts a notable influence on alignment efficiency and transcript quantification, which in turn can alter the composition of DEGs. Outcomes are also sensitive to the statistical thresholds employed; altering the cutoffs for adjusted p-value or log2 fold change may shift the balance between sensitivity and specificity in DEG detection. Notably, the studies included here utilized different strains of *Xf* for infection, which likely contributed to heterogeneity in host response. A more unified experimental design using a single, well-characterized strain could enhance the resolution and reliability of conserved transcriptional signals.

To build upon the current findings, future research should prioritize the functional validation of identified hub genes, particularly *BAK1*,* PED1*,* BAS*,* PIC30*, and *WRKY33* which have emerged as central components in the plant immune response network. Techniques such as quantitative real-time PCR and RNA interference can be employed to evaluate their roles in resistance *to Xf*. To directly assess the contribution of these genes to resistance against *Xf*, CRISPR/Cas9-based gene editing represents a powerful and precise approach for targeted functional genomics. The application of CRISPR/Cas9 in plant systems has been successfully demonstrated in numerous crops for validating defense-related genes, highlighting its potential utility in this context [27, 54]. By generating knockout or knock-in mutations in *BAK1*,* PED1*,* BAS*,* PIC30*, and *WRKY33* researchers can investigate the phenotypic consequences of gene disruption in planta, providing definitive evidence for their roles in *Xf* resistance or susceptibility.

## Final remarks

The analysis identified 18 differentially expressed genes (DEGs) linked to resistance against *Xylella fastidiosa*, including *BAK1*,* PED1*,* BAS*,* PIC30*, *WRKY33*, *VEIN PATTERNING 1* and WRKY40, pivotal for immune responses and plant resilience. Their consistent expression across host plants suggests a shared resistance network. Notably, the protein-protein interactions among these DEGs form a complex regulatory framework crucial for understanding the plant’s defense mechanisms. This network represents a sort of in silico scenario, where computational models can predict and validate the functional roles of these genes in immunity. These findings are instrumental for future research on plant resistance, guiding the functional validation of these genes and informing breeding programs for resistant cultivars, especially in olives and grapes. Future studies should investigate the roles of these genes in immunity and their interactions within signaling networks. Genomic technologies like CRISPR/Cas9 hold promise in the future to enhance resistance traits, and combining these with traditional breeding is essential for addressing *Xylella fastidiosa* challenges. Ultimately, integrating genomic data with breeding strategies will help develop resilient crops, enhancing food security and promoting sustainable agriculture.

## Supplementary Information

Supplementary Material 1.

Supplementary Material 2.

Supplementary Material 3.

Supplementary Material 4.

Supplementary Material 5.

Supplementary Material 6.

Supplementary Material 7.

Supplementary Material 8.

Supplementary Material 9.

Supplementary Material 10.

Supplementary Material 11.

Supplementary Material 12.

Supplementary Material 13.

Supplementary Material 14.

## Data Availability

All data generated or analysed during this study are included in this published article and its supplementary information files.

## References

[1] Abou Kubaa R, Giampetruzzi A, Altamura G, Saponari M, Saldarelli P. Infections of the *Xylella fastidiosa* subsp. *Pauca* strain de Donno in alfalfa (*Medicago sativa*) elicits an overactive immune response. Plants. 2019;8(9): 335. 10.3390/plants8090335. 31500293 10.3390/plants8090335 PMC6784145

[2] Alméras E, Stolz S, Vollenweider S, Reymond P, Mène-Saffrané L, Farmer EE. Reactive electrophile species activate defense gene expression in *Arabidopsis*. Plant J. 2003;34(2):205–16. 10.1046/j.1365-313x.2003.01718.x. 12694595 10.1046/j.1365-313x.2003.01718.x

[3] Bader GD, Hogue CW. An automated method for finding molecular complexes in large protein interaction networks. BMC Bioinformatics. 2003;4:2. 10.1186/1471-2105-4-2. PMID: 12525261; PMCID: PMC149346. 12525261 10.1186/1471-2105-4-2 PMC149346

[4] Bajocco S, Raparelli E, Bregaglio S. Assessing the driving role of the anthropogenic landscape on the distribution of the *Xylella fastidiosa*-driven olive quick decline syndrome in Apulia (Italy). Sci Total Environ. 2023;896: 165231. 10.1016/j.scitotenv.2023.165231. 37392876 10.1016/j.scitotenv.2023.165231

[5] Baldi P, La Porta N. *Xylella fastidiosa*: host range and advance in molecular identification techniques. Front Plant Sci. 2017;8:944. 10.3389/fpls.2017.00944. PMID: 28642764; PMCID: PMC5462928. 28642764 10.3389/fpls.2017.00944 PMC5462928

[6] Benny J, Marchese A, Giovino A, Marra FP, Perrone A, Caruso T, Martinelli F. Gaining insight into exclusive and common transcriptomic features linked to drought and salinity responses across fruit tree crops. Plants. 2020;9(9): 1059. 10.3390/plants9091059. 32825043 10.3390/plants9091059 PMC7570245

[7] Bernal AJ, Jensen JK, Harholt J, Sørensen S, Moller I, Blaukopf C, Johansen B, de Lotto R, Pauly M, Scheller HV, Willats WG. Disruption of ATCSLD5 results in reduced growth, reduced xylan and homogalacturonan synthase activity and altered xylan occurrence in *Arabidopsis*. *Plant J*. 2007;52(5):791–802. 10.1111/j.1365-313X.2007.03281.x. PMID: 17892446. 10.1111/j.1365-313X.2007.03281.x 17892446

[8] Broekaert WF, Delauré SL, De Bolle MF, Cammue BP. The role of ethylene in host-pathogen interactions. *Annu Rev Phytopathol*. 2006;44:393–416. 10.1146/annurev.phyto.44.070505.143440. PMID: 16602950. 10.1146/annurev.phyto.44.070505.143440 16602950

[9] Cardone G, Digiaro M, Djelouah K, Frem M, Rota C, Lenders A, Fucilli V. Socio-economic risks posed by a new plant disease in the mediterranean basin. Diversity. 2022;14(11):975. 10.3390/d14110975.

[10] Castaño-Miquel L, Mas A, Teixeira I, Seguí J, Perearnau A, Thampi BN, Schapire AL, Rodrigo N, La Verde G, Manrique S, Coca M, Lois LM. SUMOylation inhibition mediated by disruption of SUMO E1-E2 interactions confers plant susceptibility to necrotrophic fungal pathogens. Mol Plant. 2017;10(5):709–20. 10.1016/j.molp.2017.01.007. 28343913 10.1016/j.molp.2017.01.007

[11] Castro C, DiSalvo B, Roper MC. *Xylella fastidiosa*: a reemerging plant pathogen that threatens crops globally. PLoS Pathog. 2021;17(9). 10.1371/journal.ppat.1009813. 10.1371/journal.ppat.1009813 PMC8428566 34499674

[14] Chen J. Bringing it in: a transporter of extracellular amino acids for regulation of plant immunity. Plant Physiol. 2022;190(1):190–2. 10.1093/plphys/kiac310. 35751611 10.1093/plphys/kiac310 PMC9434176

[13] Chen H, De La Fuente L. Calcium transcriptionally regulates movement, recombination and other functions of *Xylella fastidiosa* under constant flow inside microfluidic chambers. *Microb Biotechnol*. 2020 Mar;13(2):548-561. Epub 2019 Nov 14. PMID: 31729188; PMCID: PMC7017821. 10.1111/1751-7915.13512 10.1111/1751-7915.13512 PMC7017821 31729188

[15] Chen K, Gao J, Sun S, Zhang Z, Yu B, Li J, Xie C, Li G, Wang P, Song CP, Bressan RA, Hua J, Zhu JK, Zhao Y. BONZAI proteins control global osmotic stress responses in plants. *Curr Biol*. 2020;30(24):4815–25.e4. PMID: 33035480. 10.1016/j.cub.2020.09.016 10.1016/j.cub.2020.09.016 33035480

[12] Chen C, Zhang Y, Cai J, Qiu Y, Li L, Gao C, Gao Y, Ke M, Wu S, Wei C, Chen J, Xu T, Friml J, Wang J, Li R, Chao D, Zhang B, Chen X, Gao Z. Multi-copper oxidases SKU5 and SKS1 coordinate cell wall formation using apoplastic redox-based reactions in roots. Plant Physiol. 2023;192(3):2243–60. 10.1093/plphys/kiad207. 37010107 10.1093/plphys/kiad207 PMC10315306

[16] Chin CH, Chen SH, Wu HH, Ho CW, Ko MT, Lin CY. CytoHubba: identifying hub objects and sub-networks from complex interactome. BMC Syst Biol. 2014;8(Suppl 4): S11. 10.1186/1752-0509-8-S4-S11. 25521941 10.1186/1752-0509-8-S4-S11 PMC4290687

[17] Chinchilla D, Zipfel C, Robatzek S, Kemmerling B, Nürnberger T, Jones JD, Felix G, Boller T. A flagellin-induced complex of the receptor FLS2 and BAK1 initiates plant defence. Nature. 2007;448(7152):497–500. 10.1038/nature05999. 17625569 10.1038/nature05999

[18] Delgado C, Mora-Poblete F, Ahmar S, Chen JT, Figueroa CR. Jasmonates and plant salt stress: molecular players, physiological effects, and improving tolerance by using genome-associated tools. Int J Mol Sci. 2021;22(6): 3082. 10.3390/ijms22063082. 33802953 10.3390/ijms22063082 PMC8002660

[19] Denancé N, Briand M, Gaborieau R, Gaillard S, Jacques MA. Identification of genetic relationships and subspecies signatures in *Xylella fastidiosa*. BMC Genomics. 2019;20(1):239. 10.1186/s12864-019-5565-9. PMID: 30909861; PMCID: PMC6434890. 30909861 10.1186/s12864-019-5565-9 PMC6434890

[20] Dielen AS, Badaoui S, Candresse T, German-Retana S. The ubiquitin/26S proteasome system in plant-pathogen interactions: a never-ending hide-and-seek game. *Mol Plant Pathol*. 2010;11(2):293–308. 2009.00596.x. Erratum in: *Mol Plant Pathol*. 2011;12(1):103. PMID: 20447278; PMCID: PMC6640532. 10.1111/j.1364-3703.2009.00596.x PMC6640532 20447278

[21] Eh TJ, Jiang Y, Jiang M, Li J, Lei P, Ji X, Kim HI, Zhao X, Meng F. The role of trehalose metabolism in plant stress tolerance. *J Adv Res*. 2024;S2090-1232(24)00603-9. 10.1016/j.jare.2024.12.025. PMID: 39708962.10.1016/j.jare.2024.12.02539708962

[22] Fan J, Hill L, Crooks C, Doerner P, Lamb C. Abscisic acid has a key role in modulating diverse plant-pathogen interactions. Plant Physiol. 2009;150(4):1750–61. 10.1104/pp.109.137943. 19571312 10.1104/pp.109.137943 PMC2719142

[23] Federici MT, Marcondes JA, Picchi SC, Stuchi ES, Fadel AL, Laia ML, Lemos MVF, Macedo Lemos EG. Xylella fastidiosa: An in vivo system to study possible survival strategies within citrus xylem vessels based on global gene expression analysis. Electron J Biotechnol [Internet]. 2012 May 11 [cited 2025 Jul 31];15(3). https://www.ejbiotechnology.info/index.php/ejbiotechnology/article/view/v15n3-4

[24] Fernández FD, Arias-Giraldo LF, Tolocka PA, et al. Phylogenomic analysis of *Xylella fastidiosa* subsp. Pauca strains from Olive and almond trees in Argentina. Eur J Plant Pathol. 2025;171:593–602. 10.1007/s10658-024-02969-z.

[25] Fu ZQ, Yan S, Saleh A, Wang W, Ruble J, Oka N, Mohan R, Spoel SH, Tada Y, Zheng N, Dong X. NPR3 and NPR4 are receptors for the immune signal Salicylic acid in plants. Nature. 2012;486(7402):228–32. 10.1038/nature11162. 22699612 10.1038/nature11162 PMC3376392

[27] Gao C. Genome engineering for crop improvement and future agriculture. Cell. 2021;184(6):1621–35. 10.1016/j.cell.2021.01.005. 33581057 10.1016/j.cell.2021.01.005

[26] Gao C, Zhao Y, Wang W, Zhang B, Huang X, Wang Y, Tang D. BRASSINOSTEROID-SIGNALING KINASE 1 modulates OPEN STOMATA 1 phosphorylation and contributes to stomatal closure and plant immunity. Plant J. 2024;120(1):45–59. 10.1111/tpj.16968. 39126292 10.1111/tpj.16968

[28] García Bossi J, Kumar K, Barberini ML, Domínguez GD, Rondón Guerrero YDC, Marino-Buslje C et al. The role of P-type IIA and P-type IIB Ca²⁺-ATPases in plant development and growth. *J Exp Bot*. 2020;71(4):1239-48. 10.1093/jxb/erz521. PMID: 31740935. 10.1093/jxb/erz521 31740935

[29] Gasperini D, Howe GA. Phytohormones in a universe of regulatory metabolites: lessons from jasmonate. Plant Physiol. 2024;195(1):135–54. 10.1093/plphys/kiae045. 38290050 10.1093/plphys/kiae045 PMC11060663

[30] Germain V, Rylott EL, Larson TR, et al. Requirement for 3-ketoacyl-CoA thiolase-2 in peroxisome development, fatty acid beta-oxidation and breakdown of triacylglycerol in lipid bodies of *Arabidopsis* seedlings. Plant J. 2001;28(1):1–12. 10.1046/j.1365-313x.2001.01095.x. 11696182 10.1046/j.1365-313x.2001.01095.x

[31] Giampetruzzi A, Morelli M, Saponari M, Loconsole G, Chiumenti M, Boscia D, et al. Transcriptome profiling of two Olive cultivars in response to infection by the CoDiRO strain of *Xylella fastidiosa* subsp. Pauca. BMC Genomics. 2016;17:475. 10.1186/s12864-016-2833-9. PMID: 27350531; PMCID: PMC4924284. 27350531 10.1186/s12864-016-2833-9 PMC4924284

[32] Greenwood JR, Williams SJ. Guarding the central regulator of extracellular perception in plants - a job for two. Cell Host Microbe. 2022;30(12):1657–9. 10.1016/j.chom.2022.11.008. 36521441 10.1016/j.chom.2022.11.008

[33] Gupta N, Verma V. Next-generation sequencing and its application: empowering in public health beyond reality. In: de Nogueira MA, Simoes L, editors. Emerging and Re-emerging diseases: detection and control. Singapore: Springer; 2019. 10.1007/978-981-13-8844-6_15.

[34] Hashimoto K, Kudla J. Calcium decoding mechanisms in plants. Biochimie. 2011;93(12):2054–9. 10.1016/j.biochi.2011.05.019. 21658427 10.1016/j.biochi.2011.05.019

[35] Holmes DR, Grubb LE, Monaghan J. The jasmonate receptor COI1 is required for AtPep1-induced immune responses in *Arabidopsis Thaliana*. BMC Res Notes. 2018;11(1):555. 10.1186/s13104-018-3628-7. PMID: 30075823; PMCID: PMC6076402. 30075823 10.1186/s13104-018-3628-7 PMC6076402

[36] Hrmova M, Stratilová B, Stratilová E. Broad specific xyloglucan:xyloglucosyl transferases are formidable players in the re-modelling of plant cell wall structures. Int J Mol Sci. 2022;23(3): 1656. 10.3390/ijms23031656. 35163576 10.3390/ijms23031656 PMC8836008

[37] Ichihara H, Yamada M, Kohara M, Hirakawa H, Ghelfi A, Tamura T, Isobe SN. Plant GARDEN: a portal website for cross-searching between different types of genomic and genetic resources in a wide variety of plant species. BMC Plant Biol. 2023;23(1):391. 37568098 10.1186/s12870-023-04392-8 PMC10422841

[38] Janse JD, Obradovic A. *Xylella fastidiosa*: its biology, diagnosis, control, and risks. J Plant Pathol. 2010;92(1):S135–48.

[39] Jiang S, Pan L, Zhou Q, Xu W, He F, Zhang L, Gao H. Analysis of the apoplast fluid proteome during the induction of systemic acquired resistance in*Arabidopsis Thaliana*. PeerJ. 2023;11. 10.7717/peerj.16324. 10.7717/peerj.16324 PMC10592298 37876907

[40] Jun JH, Ha CM, Nam HG. Involvement of the VEP1 gene in vascular strand development in *Arabidopsis thaliana*. Plant Cell Physiol. 2002;43(3):323–30. 10.1093/pcp/pcf042. 11917087 10.1093/pcp/pcf042

[41] Kadota Y, Sklenar J, Derbyshire P, Stransfeld L, Asai S, Ntoukakis V, Jones JD, Shirasu K, Menke F, Jones A, Zipfel C. Direct regulation of the NADPH oxidase RBOHD by the PRR-associated kinase BIK1 during plant immunity. *Mol Cell*. 2014;54(1):43–55. 10.1016/j.molcel.2014.02.021. PMID: 24630626. 10.1016/j.molcel.2014.02.021 24630626

[42] Karapetyan S, Dong X. Redox and the circadian clock in plant immunity: a balancing act. Free Radic Biol Med. 2018;119:56–61. 10.1016/j.freeradbiomed.2017.12.024. 29274381 10.1016/j.freeradbiomed.2017.12.024 PMC5986284

[43] Keel BN, Lindholm-Perry AK. Recent developments and future directions in meta-analysis of differential gene expression in livestock RNA-Seq. Front Genet. 2022;13:983043. 10.3389/fgene.2022.983043. PMID: 36199583; PMCID: PMC9527320. 36199583 10.3389/fgene.2022.983043 PMC9527320

[44] Killiny N, Almeida RP. Factors affecting the initial adhesion and retention of the plant pathogen *Xylella fastidiosa* in the foregut of an insect vector. Appl Environ Microbiol. 2014;80(1):420–6. 10.1128/AEM.03156-13. PMID: 24185853; PMCID: PMC3910991. 24185853 10.1128/AEM.03156-13 PMC3910991

[46] Kim TW, Hwang JY, Kim YS, Joo SH, Chang SC, Lee JS, et al. *Arabidopsis* CYP85A2, a cytochrome P450, mediates the Baeyer-Villiger oxidation of castasterone to Brassinolide in brassinosteroid biosynthesis. Plant Cell. 2005;17(8):2397–412. 10.1105/tpc.105.033738. PMID: 16024588; PMCID: PMC1182497. 16024588 10.1105/tpc.105.033738 PMC1182497

[45] Kim GH, Lee YS, Jung JS, Koh YJ, Poulter RTM, Butler M. Genomic analyses of *Pseudomonas syringae* pv. *actinidiae* isolated in Korea suggest the transfer of the bacterial pathogen via kiwifruit pollen. J Med Microbiol. 2020;69(1):132–8. 10.1099/jmm.0.001115. 31859618 10.1099/jmm.0.001115

[47] Kole C, Muthamilarasan M, Henry R, Edwards D, Sharma R, Abberton M, et al. Application of genomics-assisted breeding for generation of climate resilient crops: progress and prospects. Front Plant Sci. 2015;6:563. 10.3389/fpls.2015.00563. PMID: 26322050; PMCID: PMC4531421. 26322050 10.3389/fpls.2015.00563 PMC4531421

[48] Kong L, Liu Y, Zhi P, Wang X, Xu B, Gong Z, Chang C. Origins and evolution of cuticle biosynthetic machinery in land plants. Plant Physiol. 2020;184(4):1998–2010. 10.1104/pp.20.00913. PMID: 32934149; PMCID: PMC7723097. 32934149 10.1104/pp.20.00913 PMC7723097

[49] Krugner R, Ledbetter CA. Rootstock effects on almond leaf scorch disease incidence and severity. Plant Dis. 2016;100(8):1617–21. 10.1094/PDIS-01-16-0125-RE. 30686222 10.1094/PDIS-01-16-0125-RE

[50] Kusch S, Panstruga R. Mlo-based resistance: an apparently universal weapon to defeat powdery mildew disease. Mol Plant Microbe Interact. 2017;30(3):179–89. 10.1094/MPMI-12-16-0255-CR. 28095124 10.1094/MPMI-12-16-0255-CR

[51] La Notte P, Saponari M, Mousavi S, Mariotti R, Abou Kubaa R, Nikbakht R, et al. A survey in natural Olive resources exposed to high inoculum pressure indicates the presence of traits of resistance to *Xylella fastidiosa* in leccino offspring. Front Plant Sci. 2024;15:1457831. 10.3389/fpls.2024.1457831. PMID: 39403622; PMCID: PMC11471571. 39403622 10.3389/fpls.2024.1457831 PMC11471571

[52] Landa BB, Saponari M, Feitosa-Junior OR, et al. *Xylella fastidiosa*’s relationships: the bacterium, the host plants, and the plant microbiome. New Phytol. 2022;234(5):1598–605. 10.1111/nph.18089. 35279849 10.1111/nph.18089

[53] Li J, Lease KA, Tax FE, Walker JC. BRS1, a serine carboxypeptidase, regulates BRI1 signaling in *Arabidopsis Thaliana*. Proc Natl Acad Sci U S A. 2001;98(10):5916–21. 10.1073/pnas.091065998. 11320207 10.1073/pnas.091065998 PMC33313

[57] Li Y, Li S, Bi D, Cheng YT, Li X, Zhang Y. SRFR1 negatively regulates plant NB-LRR resistance protein accumulation to prevent autoimmunity. PLoS Pathog. 2010;6(9):e1001111. 10.1371/journal.ppat.1001111. PMID: 20862316; PMCID: PMC2940742. 20862316 10.1371/journal.ppat.1001111 PMC2940742

[54] Li J, Meng X, Zong Y, Chen K, Zhang H, Liu J, Li J, Gao C. Gene replacements and insertions in rice by intron targeting using CRISPR-Cas9. *Nat Plants*. 2016;2:16139. 10.1038/nplants.2016.139. PMID: 27618611. 10.1038/nplants.2016.139 27618611

[55] Li N, Han X, Feng D, Yuan D, Huang LJ. Signaling crosstalk between salicylic acid and ethylene/jasmonate in plant defense: do we understand what they are whispering? Int J Mol Sci. 2019;20(3): 671. 10.3390/ijms20030671. 30720746 10.3390/ijms20030671 PMC6387439

[56] Li P, Zhao L, Qi F, Htwe NMPS, Li Q, Zhang D et al. The receptor-like cytoplasmic kinase RIPK regulates broad-spectrum ROS signaling in multiple layers of plant immune system. *Mol Plant*. 2021;14(10):1652–67. 10.1016/j.molp.2021.06.010. PMID: 34129947. 10.1016/j.molp.2021.06.010 34129947

[58] Li-Beisson Y, Shorrosh B, Beisson F, Andersson MX, Arondel V, Bates PD, et al. Acyl-lipid metabolism. Arabidopsis Book. 2013;11: e0161. 10.1199/tab.0161. 23505340 10.1199/tab.0161 PMC3563272

[59] Limonta M, Romanowsky S, Olivari C, Bonza MC, Luoni L, Rosenberg A, et al. ACA12 is a deregulated isoform of plasma membrane Ca²⁺-ATPase of *Arabidopsis Thaliana*. Plant Mol Biol. 2014;84(4–5):387–97. 10.1007/s11103-013-0138-9. PMID: 24101142; PMCID: PMC4104672. 24101142 10.1007/s11103-013-0138-9 PMC4104672

[60] Liu X, Yue Y, Li B, Nie Y, Li W, Wu WH, Ma L. A G protein-coupled receptor is a plasma membrane receptor for the plant hormone abscisic acid. Science. 2007;315(5819):1712–6. 10.1126/science.1135882. 17347412 10.1126/science.1135882

[61] Lu J, Liang W, Zhang N, van Wersch S, Li X. HSP90 contributes to chs3-2D-mediated autoimmunity. Front Plant Sci. 2022;13:888449. 10.3389/fpls.2022.888449. PMID: 35720559; PMCID: PMC9204091. 35720559 10.3389/fpls.2022.888449 PMC9204091

[62] Luo N, Wang Y, Liu Y, Wang Y, Guo Y, Chen C, et al. 3-ketoacyl-CoA synthase 19 contributes to the biosynthesis of seed lipids and cuticular wax in *Arabidopsis* and abiotic stress tolerance. Plant Cell Environ. 2024;47(12):4599–614. 10.1111/pce.15054. 39041727 10.1111/pce.15054

[63] Ma C, Liu Y, Bai B, Han Z, Tang J, Zhang H, Yaghmaiean H, Zhang Y, Chai J. Structural basis for BIR1-mediated negative regulation of plant immunity. Cell Res. 2017;27(12):1521–4. 10.1038/cr.2017.123. PMID: 28961230; PMCID: PMC5717402. 28961230 10.1038/cr.2017.123 PMC5717402

[64] Ma J, Morel JB, Riemann M, Nick P. Jasmonic acid contributes to rice resistance against *Magnaporthe oryzae*. BMC Plant Biol. 2022;22(1):601. 10.1186/s12870-022-03948-4. PMID: 36539712; PMCID: PMC9764487. 36539712 10.1186/s12870-022-03948-4 PMC9764487

[65] Macioszek VK, Jęcz T, Ciereszko I, Kononowicz AK. Jasmonic acid as a mediator in plant response to necrotrophic fungi. Cells. 2023;12(7):1027. 10.3390/cells12071027. PMID: 37048100; PMCID: PMC10093439. 37048100 10.3390/cells12071027 PMC10093439

[66] Martinelli F, Marchese A, Giovino A, Marra FP, Della Noce I, Caruso T, et al.. Front Plant Sci. 2019;9:2007. 10.3389/fpls.2018.02007. PMID: 30713547; PMCID: PMC6345699. 10.3389/fpls.2018.02007 PMC6345699 30713547

[67] Mendes JS, Santiago AS, Toledo MA, Horta MA, de Souza AA, Tasic L, et al. In vitro determination of extracellular proteins from *Xylella fastidiosa*. Front Microbiol. 2016;7:2090. 10.3389/fmicb.2016.02090. PMID: 28082960; PMCID: PMC5183587. 28082960 10.3389/fmicb.2016.02090 PMC5183587

[68] Miao G, Han J, Liu C, Liu J, Wang C, Wang S. PDR6-mediated camalexin efflux and disease resistance are regulated through direct phosphorylation by the kinases OXI1 and AGC2-2. BioRxiv [Preprint]. 2022. 10.1101/2022.02.08.479400.36451890

[69] Mitre LK, Teixeira-Silva NS, Rybak K, Magalhães DM, de Souza-Neto RR, Robatzek S, Zipfel C, de Souza AA. The *Arabidopsis* immune receptor EFR increases resistance to the bacterial pathogens *Xanthomonas* and *Xylella* in transgenic sweet orange. Plant Biotechnol J. 2021;19(7):1294–6. 10.1111/pbi.13629. 33991397 10.1111/pbi.13629 PMC8313127

[70] Mittler R, Zandalinas SI, Fichman Y, Van Breusegem F. Reactive oxygen species signalling in plant stress responses. Nat Rev Mol Cell Biol. 2022;23(10):663–79. 10.1038/s41580-022-00499-2. 35760900 10.1038/s41580-022-00499-2

[71] Moll L, Baró A, Montesinos L, Badosa E, Bonaterra A, Montesinos E. Induction of defense responses and protection of almond plants against *Xylella fastidiosa* by endotherapy with a bifunctional peptide. Phytopathology. 2022;112(9):1907–16. 10.1094/PHYTO-12-21-0525-R. 35384723 10.1094/PHYTO-12-21-0525-R

[72] Monaghan J, Xu F, Xu S, Zhang Y, Li X. Two putative RNA-binding proteins function with unequal genetic redundancy in the MOS4-associated complex. Plant Physiol. 2010;154(4):1783–93. 10.1104/pp.110.158931. 20943852 10.1104/pp.110.158931 PMC2996007

[73] Morales-Cruz A, Aguirre-Liguori J, Massonnet M, et al. Multigenic resistance to *Xylella fastidiosa* in wild grapes (*Vitis* spp.) and its implications within a changing climate. Commun Biol. 2023;6: 580. 10.1038/s42003-023-04938-4. 37253933 10.1038/s42003-023-04938-4 PMC10229667

[74] Novelli S, Gismondi A, Di Marco G, Canuti L, Nanni V, Canini A. Plant defense factors involved in *Olea europaea* resistance against *Xylella fastidiosa* infection. J Plant Res. 2019;132(3):439–55. 10.1007/s10265-019-01108-8. 30993555 10.1007/s10265-019-01108-8

[75] Patra N, Hariharan S, Gain H, Maiti MK, Das A, Banerjee J. Typical but delicate Ca + + re: dissecting the essence of calcium signaling network as a robust response coordinator of versatile abiotic and biotic stimuli in plants. Front Plant Sci. 2021;12:752246. 10.3389/fpls.2021.752246. PMID: 34899779; PMCID: PMC8655846. 34899779 10.3389/fpls.2021.752246 PMC8655846

[76] Pavan S, Vergine M, Nicolì F, Sabella E, Aprile A, Negro C, Fanelli V, Savoia MA, Montilon V, Susca L, Delvento C, Lotti C, Nigro F, Montemurro C, Ricciardi L, De Bellis L, Luvisi A. Screening of olive biodiversity defines genotypes potentially resistant to *Xylella fastidiosa*. Front Plant Sci. 2021;12:723879. 10.3389/fpls.2021.723879. PMID: 34484283; PMCID: PMC8415753. 10.3389/fpls.2021.723879 PMC8415753 34484283

[77] Pieterse CM, Van der Does D, Zamioudis C, Leon-Reyes A, Van Wees SC. Hormonal modulation of plant immunity. Annu Rev Cell Dev Biol. 2012;28:489–521. 10.1146/annurev-cellbio-092910-154055. 22559264 10.1146/annurev-cellbio-092910-154055

[78] Purcell AH, Hopkins DL. Fastidious xylem-limited bacterial plant pathogens. Annu Rev Phytopathol. 1996;34:131–51. 10.1146/annurev.phyto.34.1.131. 15012538 10.1146/annurev.phyto.34.1.131

[79] Rahmati Ishka M, Brown E, Rosenberg A, Romanowsky S, Davis JA, Choi WG, Harper JF. *Arabidopsis* Ca2+-ATPases 1, 2, and 7 in the Endoplasmic reticulum contribute to growth and pollen fitness. Plant Physiol. 2021;185(4):1966–85. 10.1093/plphys/kiab021. PMID: 33575795; PMCID: PMC8133587. 33575795 10.1093/plphys/kiab021 PMC8133587

[81] Rapicavoli JN, Blanco-Ulate B, Muszyński A, Figueroa-Balderas R, Morales-Cruz A, Azadi P, Dobruchowska JM, Castro C, Cantu D, Roper MC. Lipopolysaccharide O-antigen delays plant innate immune recognition of *Xylella fastidiosa*. Nat Commun. 2018;9(1):390. 10.1038/s41467-018-02861-5. PMID: 29374171; PMCID: PMC5786101. 29374171 10.1038/s41467-018-02861-5 PMC5786101

[80] Rapicavoli J, Ingel B, Blanco-Ulate B, Cantu D, Roper C. *Xylella fastidiosa*: an examination of a re-emerging plant pathogen. Mol Plant Pathol. 2018a;19(4):786–800. PMID: 28742234; PMCID: PMC6637975. 10.1111/mpp.12585 10.1111/mpp.12585 PMC6637975 28742234

[82] Rizwan HM, Shaozhong F, Li X, Arshad MB, Yousef AF, Chenglong Y, Shi M, Jaber MYM, Anwar M, Hu SY, Yang Q, Sun K, Ahmed MAA, Min Z, Oelmüller R, Zhimin L, Chen F. Genome-wide identification and expression profiling of KCS gene family in passion fruit (*Passiflora edulis*) under *Fusarium Kyushuense* and drought stress conditions. Front Plant Sci. 2022;13:872263. 10.3389/fpls.2022.872263. PMID: 35548275; PMCID: PMC9081883. 35548275 10.3389/fpls.2022.872263 PMC9081883

[83] Rodrigues CM, de Souza AA, Takita MA, Kishi LT, Machado MA. RNA-seq analysis of *Citrus reticulata* in the early stages of *Xylella fastidiosa* infection reveals auxin-related genes as a defense response. BMC Genomics. 2013;14:676. 10.1186/1471-2164-14-676. PMID: 24090429; PMCID: PMC3852278. 24090429 10.1186/1471-2164-14-676 PMC3852278

[84] Saponari M, Boscia D, Nigro F, Martelli GP. Identification of DNA sequences related to *Xylella fastidiosa* in oleander, almond, and olive trees exhibiting leaf scorch symptoms in Apulia (Southern Italy). J Plant Pathol. 2013;95(3):659–68.

[85] Saponari M, Loconsole G, Cornara D, Yokomi RK, De Stradis A, Boscia D, Bosco D, Martelli GP, Krugner R, Porcelli F. Infectivity and transmission of *Xylella fastidiosa* by *Philaenus spumarius* (Hemiptera: Aphrophoridae) in Apulia, Italy. J Econ Entomol. 2014;107(4):1316–9. 10.1603/ec14142. 25195417 10.1603/ec14142

[86] Savadi S, Mangalassery S, Sandesh MS. Advances in genomics and genome editing for breeding next generation of fruit and nut crops. Genomics. 2021;113(6):3718–34. 10.1016/j.ygeno.2021.09.001. 34517092 10.1016/j.ygeno.2021.09.001

[87] Schaad NW, Postnikova E, Lacy G, Fatmi M, Chang CJ. *Xylella fastidiosa* subspecies: *X. fastidiosa* subsp. [correction] *fastidiosa* [correction] subsp. nov., *X. fastidiosa* subsp. *multiplex* subsp. nov., and *X. fastidiosa* subsp. *pauca* subsp. nov. Syst Appl Microbiol. 2004;27(3):290–300. 10.1078/0723-2020-00263. 15214634 10.1078/0723-2020-00263

[88] Schluepmann H, Paul M. Trehalose metabolites in *Arabidopsis*—elusive, active and central. Arabidopsis Book. 2009;7:e0122. 10.1199/tab.0122. PMID: 22303248; PMCID: PMC3243345. 22303248 10.1199/tab.0122 PMC3243345

[89] Schuenzel EL, Scally M, Stouthamer R, Nunney L. A multigene phylogenetic study of clonal diversity and divergence in North American strains of the plant pathogen *Xylella fastidiosa*. Appl Environ Microbiol. 2005;71(7):3832–9. 10.1128/AEM.71.7.3832-3839.2005. 16000795 10.1128/AEM.71.7.3832-3839.2005 PMC1169037

[90] Serio F, Imbriani G, Girelli CR, Miglietta PP, Scortichini M, Fanizzi FP. A decade after the outbreak of *Xylella fastidiosa* subsp. *Pauca* in Apulia (Southern Italy): methodical literature analysis of research strategies. Plants (Basel). 2024;13(11): 1433. 10.3390/plants13111433. 38891241 10.3390/plants13111433 PMC11175074

[91] Sertedakis M, Kotsaridis K, Tsakiri D, Mermigka G, Dominguez-Ferreras A, Ntoukakis V, Sarris PF. Expression of putative effectors of different *Xylella fastidiosa* strains triggers cell death-like responses in various Nicotiana model plants. Mol Plant Pathol. 2022;23(1):148–56. 10.1111/mpp.13147. PMID: 34628713; PMCID: PMC8659589. 34628713 10.1111/mpp.13147 PMC8659589

[92] Shah S, Chen C, Sun Y, Wang D, Nawaz T, El-Kahtany K, Fahad S. Mechanisms of nitric oxide involvement in plant-microbe interaction and its enhancement of stress resistance. Plant Stress. 2023;10:100191. 10.1016/j.stress.2023.100191.

[93] Sherman BT, Hao M, Qiu J, Jiao X, Baseler MW, Lane HC, Imamichi T, Chang W. DAVID: a web server for functional enrichment analysis and functional annotation of gene lists (2021 update). Nucleic Acids Res. 2022;50(W1):W216–221. 10.1093/nar/gkac194. 35325185 10.1093/nar/gkac194 PMC9252805

[94] Silverstein KA, Moskal WA Jr, Wu HC, Underwood BA, Graham MA, Town CD, VandenBosch KA. Small cysteine-rich peptides resembling antimicrobial peptides have been under-predicted in plants. Plant J. 2007;51(2):262–80. 10.1111/j.1365-313X.2007.03136.x. 10.1111/j.1365-313X.2007.03136.x 17565583

[95] Singh D, Tripathi A, Mitra R, Bhati J, Rani V, Taunk J, Kumar S, Singh S, Verma P. Genome-wide identification of MATE and ALMT genes and their expression profiling in mungbean (*Vigna radiata* L.) under aluminium stress. *Ecotoxicol Environ Saf.* 2024;280:116558. 10.1016/j.ecoenv.2024.116558. PMID: 38850702. 10.1016/j.ecoenv.2024.116558 38850702

[96] Song JT, Lu H, McDowell JM, Greenberg JT. A key role for ALD1 in activation of local and systemic defenses in *Arabidopsis*. Plant J. 2004;40(2):200–12. 10.1111/j.1365-313X.2004.02200.x. 15447647 10.1111/j.1365-313X.2004.02200.x

[97] Staswick PE, Tiryaki I, Rowe ML. Jasmonate response locus JAR1 and several related *Arabidopsis* genes encode enzymes of the firefly luciferase superfamily that show activity on jasmonic, salicylic, and indole-3-acetic acids in an assay for adenylation. Plant Cell. 2002;14(6):1405–15. 10.1105/tpc.000885. 12084835 10.1105/tpc.000885 PMC150788

[98] Stefanowicz K, Lannoo N, Zhao Y, Eggermont L, Van Hove J, Al Atalah B, Van Damme EJ. Glycan-binding F-box protein from *Arabidopsis Thaliana* protects plants from *Pseudomonas syringae* infection. BMC Plant Biol. 2016;16(1):213. 10.1186/s12870-016-0905-2. PMID: 27716048; PMCID: PMC5050601. 27716048 10.1186/s12870-016-0905-2 PMC5050601

[99] Sun CW, Callis J. Independent modulation of *Arabidopsis thaliana* polyubiquitin mrnas in different organs and in response to environmental changes. Plant J. 1997;11(5):1017–27. 10.1046/j.1365-313X.1997.11051017.x. 9193073 10.1046/j.1365-313x.1997.11051017.x

[100] Szklarczyk D, Kirsch R, Koutrouli M, Nastou K, Mehryary F, Hachilif R, Barshir R, Evangelidis T, Jäger S, Cook H, McDermott J, Ochoa D, Ezkurdia I, Kuhn M, Jensen LJ, von Mering C, Bork P, Kuhn M. The STRING database in 2023: protein-protein association networks and functional enrichment analyses for any sequenced genome of interest. Nucleic Acids Res. 2023;51(D1):D638–646. 10.1093/nar/gkac1000. PMID: 36370105; PMCID: PMC9825434. 36370105 10.1093/nar/gkac1000 PMC9825434

[101] Takahashi A, Casais C, Ichimura K, Shirasu K. HSP90 interacts with RAR1 and SGT1 and is essential for RPS2-mediated disease resistance in *Arabidopsis*. Proc Natl Acad Sci U S A. 2003;100(20):11777–1182. 10.1073/pnas.2033934100. PMID: 14504384; PMCID: PMC208834. 14504384 10.1073/pnas.2033934100 PMC208834

[102] Tarrío R, Ayala FJ, Rodríguez-Trelles F. The vein patterning 1 (VEP1) gene family laterally spread through an ecological network. PLoS One. 2011;6(7):e22279. 10.1371/journal.pone.0022279. PMID: 21818306; PMCID: PMC3144213. 21818306 10.1371/journal.pone.0022279 PMC3144213

[103] Thomas E, Noar RD, Daub ME. A polyketide synthase gene cluster required for pathogenicity of *Pseudocercospora fijiensis* on banana. PLoS One. 2021;16(10):e0258981. 10.1371/journal.pone.0258981. PMID: 34705882; PMCID: PMC8550591. 34705882 10.1371/journal.pone.0258981 PMC8550591

[104] Toro-Domínguez D, Villatoro-García JA, Martorell-Marugán J, Román-Montoya Y, Alarcón-Riquelme ME, Carmona-Sáez P. A survey of gene expression meta-analysis: methods and applications. Brief Bioinform. 2021;22(2):1694–705. 10.1093/bib/bbaa019. 32095826 10.1093/bib/bbaa019

[105] Üstün S, Sheikh A, Gimenez-Ibanez S, Jones A, Ntoukakis V, Börnke F. The proteasome acts as a hub for plant immunity and is targeted by *Pseudomonas* type III effectors. Plant Physiol. 2016;172(3):1941–58. 10.1104/pp.16.00808. 27613851 10.1104/pp.16.00808 PMC5100764

[106] Verma V, Ravindran P, Kumar PP. Plant hormone-mediated regulation of stress responses. BMC Plant Biol. 2016;16:86. 10.1186/s12870-016-0771-y. PMID: 27079791; PMCID: PMC4831116. 27079791 10.1186/s12870-016-0771-y PMC4831116

[107] Vishal B, Krishnamurthy P, Kumar PP. *Arabidopsis* class II TPS controls root development and confers salt stress tolerance through enhanced hydrophobic barrier deposition. Plant Cell Rep. 2024;43(5):115. 10.1007/s00299-024-03215-w. 38613634 10.1007/s00299-024-03215-w

[108] Wang S, Tiwari SB, Hagen G, Guilfoyle TJ. Auxin response factor7 restores the expression of auxin-responsive genes in mutant *Arabidopsis* leaf mesophyll protoplasts. Plant Cell. 2005;17(7):1979–93. 10.1105/tpc.105.031096. 15923351 10.1105/tpc.105.031096 PMC1167546

[109] Wasternack C, Hause B. Jasmonates: biosynthesis, perception, signal transduction and action in plant stress response, growth and development. An update to the 2007 review in *Annals of botany*. Ann Bot. 2013;111(6):1021–58. 10.1093/aob/mct067. 23558912 10.1093/aob/mct067 PMC3662512

[110] Woloshen V, Huang S, Li X. RNA-binding proteins in plant immunity. J Pathog. 2011;2011:278697. 10.4061/2011/278697. 22567326 10.4061/2011/278697 PMC3335643

[111] Xu J, Xie J, Yan C, Zou X, Ren D, Zhang S. A chemical genetic approach demonstrates that MPK3/MPK6 activation and NADPH oxidase-mediated oxidative burst are two independent signaling events in plant immunity. Plant J. 2014;77(2):222–34. 10.1111/tpj.12382. 24245741 10.1111/tpj.12382 PMC4017028

[112] Yang DH, Hettenhausen C, Baldwin IT, Wu J. BAK1 regulates the accumulation of jasmonic acid and the levels of trypsin proteinase inhibitors in *Nicotiana attenuata*’s responses to herbivory. J Exp Bot. 2011;62(2):641–52. 10.1093/jxb/erq298. 20937731 10.1093/jxb/erq298 PMC3003809

[113] Yang L, Qiao L, Su X, Ji B, Dong C. Drought stress stimulates the terpenoid backbone and triterpenoid biosynthesis pathway to promote the synthesis of saikosaponin in *Bupleurum Chinense* DC. roots. Molecules. 2022a;27(17):5470. 10.3390/molecules27175470. PMID: 36080237; PMCID: PMC9457724. 36080237 10.3390/molecules27175470 PMC9457724

[114] Yang Z, Xia J, Hong J, Zhang C, Wei H, Ying W, Sun C, Sun Y, Mao Y, Gao Y. Structural insights into auxin recognition and efflux by *Arabidopsis* PIN1. Nature. 2022b;609(7927):611–5. 10.1038/s41586-022-05143-9. PMID: 35917925; PMCID: PMC9477737. 35917925 10.1038/s41586-022-05143-9 PMC9477737

[115] Yi SY, Lee M, Kwon SY, Kim WT, Lim YP, Kang SY. RING-type E3 ubiquitin ligases AtRDUF1 and AtRDUF2 positively regulate the expression of PR1 gene and pattern-triggered immunity. Int J Mol Sci. 2022;23(23): 14525. 10.3390/ijms232314525. 36498851 10.3390/ijms232314525 PMC9739713

[116] Yoon S, Lee WH. Assessing potential European areas of Pierce’s disease mediated by insect vectors by using spatial ensemble model. Front Plant Sci. 2023;14:1209694. 10.3389/fpls.2023.1209694. PMID: 37396635; PMCID: PMC10312007. 37396635 10.3389/fpls.2023.1209694 PMC10312007

[118] Yu Q, Chen C, Du D, Huang M, Yao J, Yu F, Zhang M, Guo Y, Gmitter FG Jr, Wang N. Reprogramming of a defense signaling pathway in rough lemon and sweet orange is a critical element of the early response to *Candidatus liberibacter Asiaticus*. Hortic Res. 2017;4:17063. 10.1038/hortres.2017.63. PMID: 29214028; PMCID: PMC5705785. 29214028 10.1038/hortres.2017.63 PMC5705785

[117] Yu H, Yan J, Du X, Hua J. Overlapping and differential roles of plasma membrane calcium ATPases in *Arabidopsis* growth and environmental responses. J Exp Bot. 2018;69(10):2693–703. 10.1093/jxb/ery073. 29506225 10.1093/jxb/ery073 PMC5920303

[119] Zaini PA, Nascimento R, Gouran H, Cantu D, Chakraborty S, Phu M, Goulart LR, Dandekar AM. Molecular profiling of pierce’s disease outlines the response circuitry of *Vitis vinifera* to *Xylella fastidiosa* infection. Front Plant Sci. 2018;9:771. 10.3389/fpls.2018.00771. PMID: 29937771; PMCID: PMC6002507. 29937771 10.3389/fpls.2018.00771 PMC6002507

[120] Zhang W, Zhao F, Jiang L, Chen C, Wu L, Liu Z. Different pathogen defense strategies in *Arabidopsis*: more than pathogen recognition. Cells. 2018;7(12):252. 10.3390/cells7120252. PMID: 30544557; PMCID: PMC6315839. 30544557 10.3390/cells7120252 PMC6315839

[121] Zhou B, Zeng L. Conventional and unconventional ubiquitination in plant immunity. Mol Plant Pathol. 2017;18(9):1313–30. 10.1111/mpp.12521. 27925369 10.1111/mpp.12521 PMC6638253

[122] Zhu A, Ibrahim JG, Love MI. Heavy-tailed prior distributions for sequence count data: removing the noise and preserving large differences. Bioinformatics. 2019;35(12):2084–92. 10.1093/bioinformatics/bty895. 30395178 10.1093/bioinformatics/bty895 PMC6581436

[123] Ziv C, Zhao Z, Gao YG, Xia Y. Multifunctional roles of plant cuticle during plant-pathogen interactions. Front Plant Sci. 2018;9: 1088. 10.3389/fpls.2018.01088. 30090108 10.3389/fpls.2018.01088 PMC6068277

