# Supplementary figures and images for: A systems biology framework integrating cross-species transcriptomics and PPI networks for Xylella fastidiosa resistance gene identification
Source: BMC Plant Biol. 2025 Aug 11;25:1062. doi: 10.1186/s12870-025-07102-8 (PMC12337395; doi:10.1186/s12870-025-07102-8)

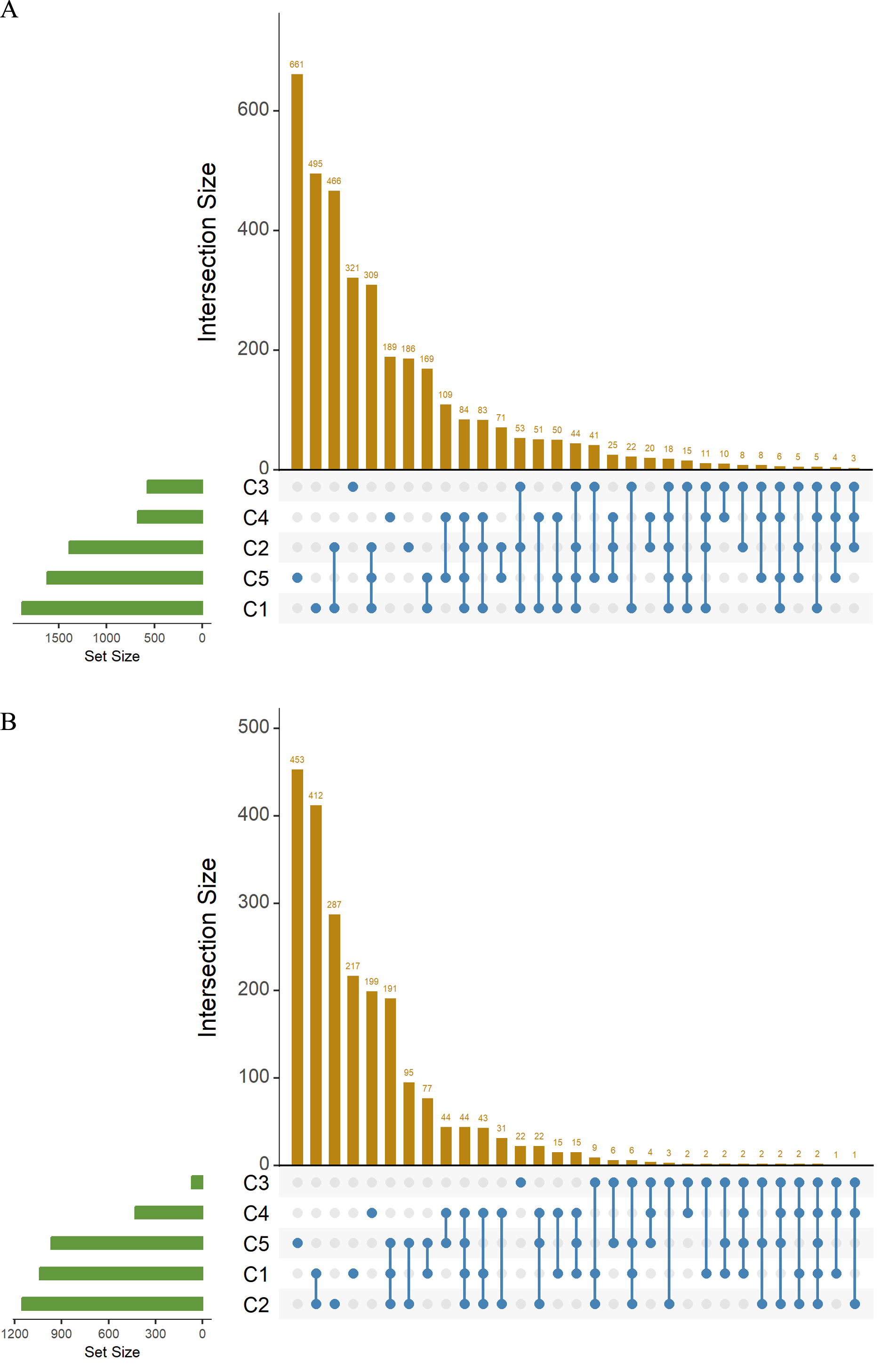

Supplement: Supplementary file 4 — Supplementary Material 4. [file 12870_2025_7102_MOESM4_ESM.tif]

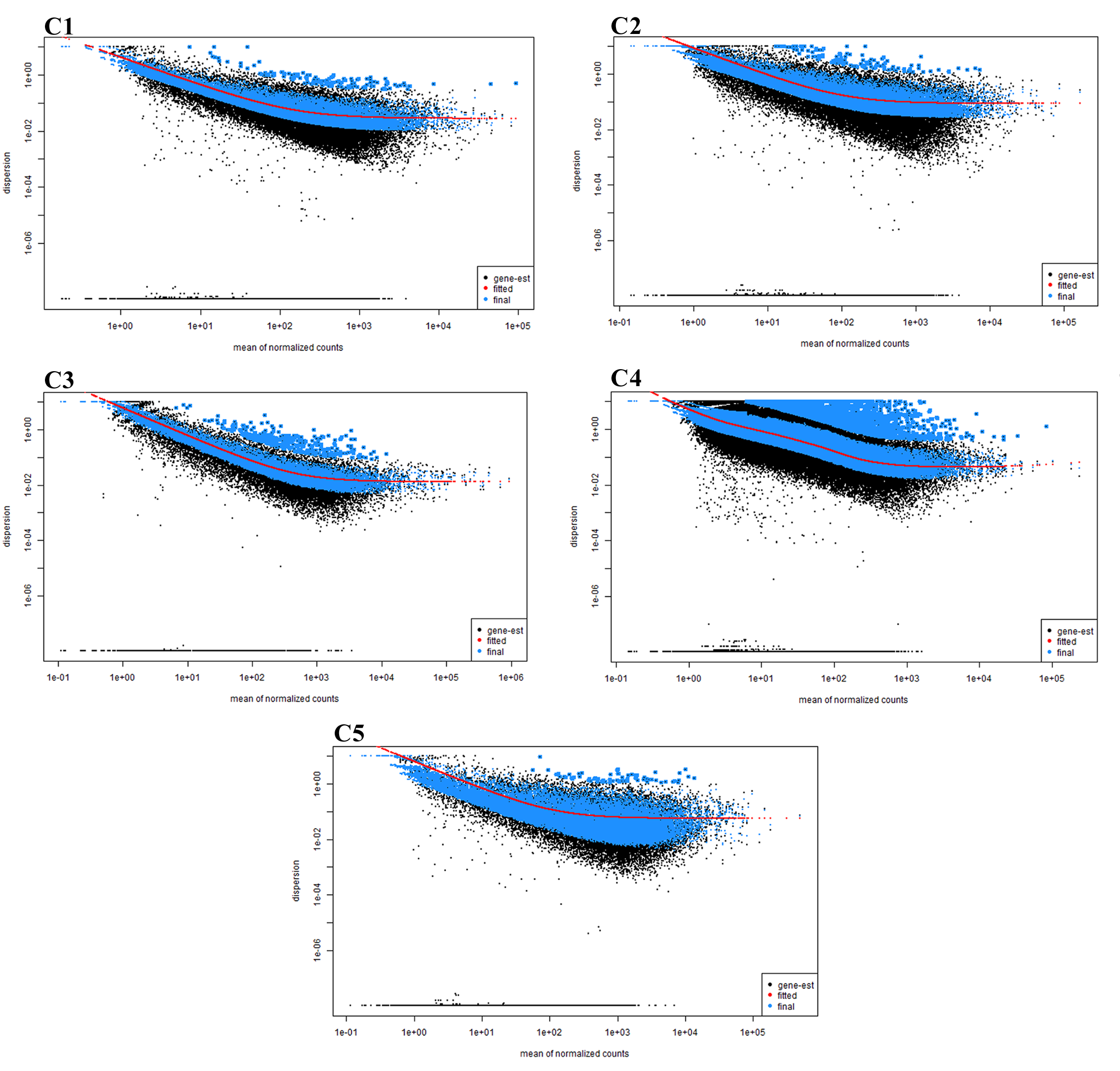

Supplement: Supplementary file 8 — Supplementary Material 8. [file 12870_2025_7102_MOESM8_ESM.tif]

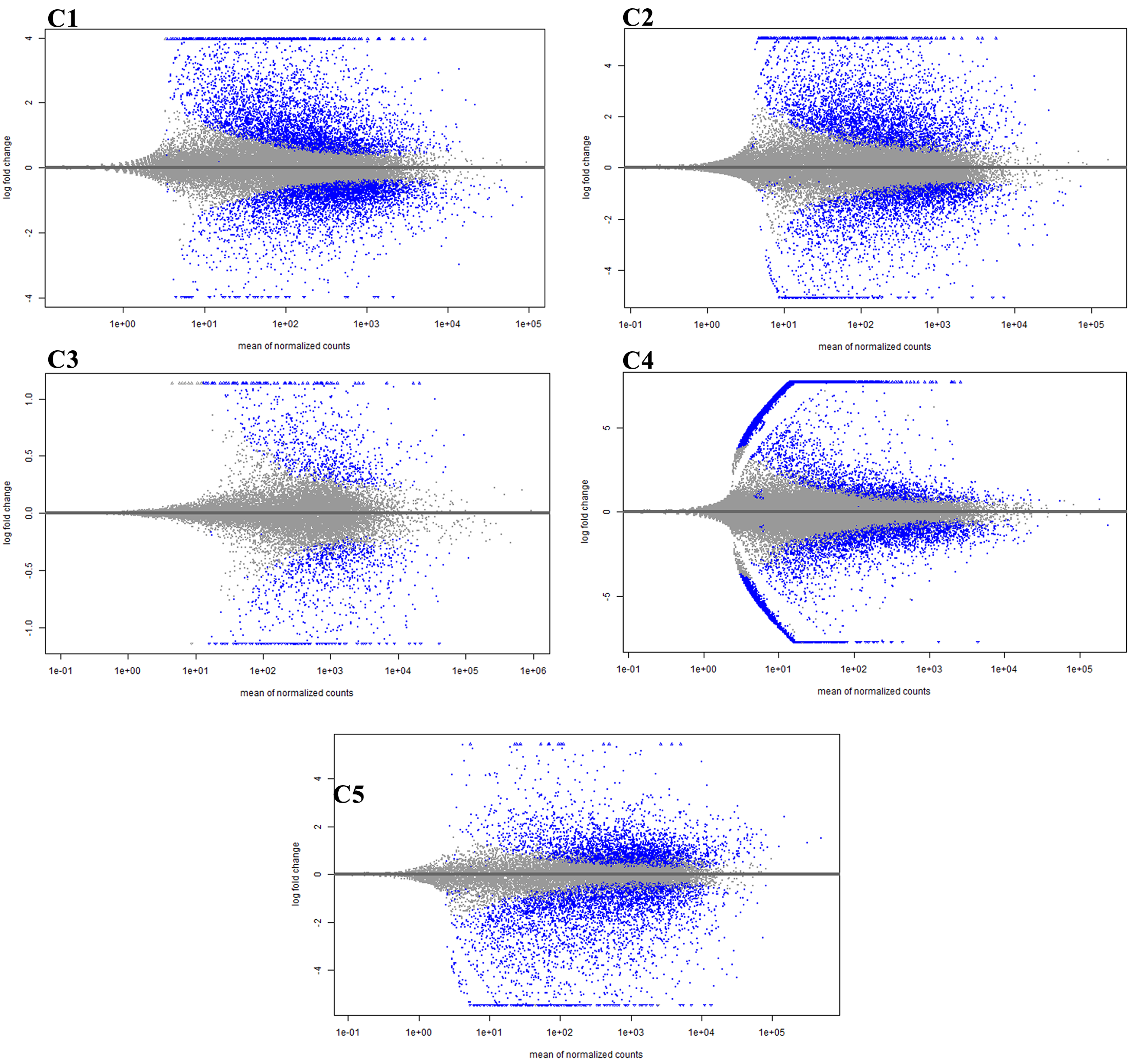

Supplement: Supplementary file 9 — Supplementary Material 9. [file 12870_2025_7102_MOESM9_ESM.tif]

C1

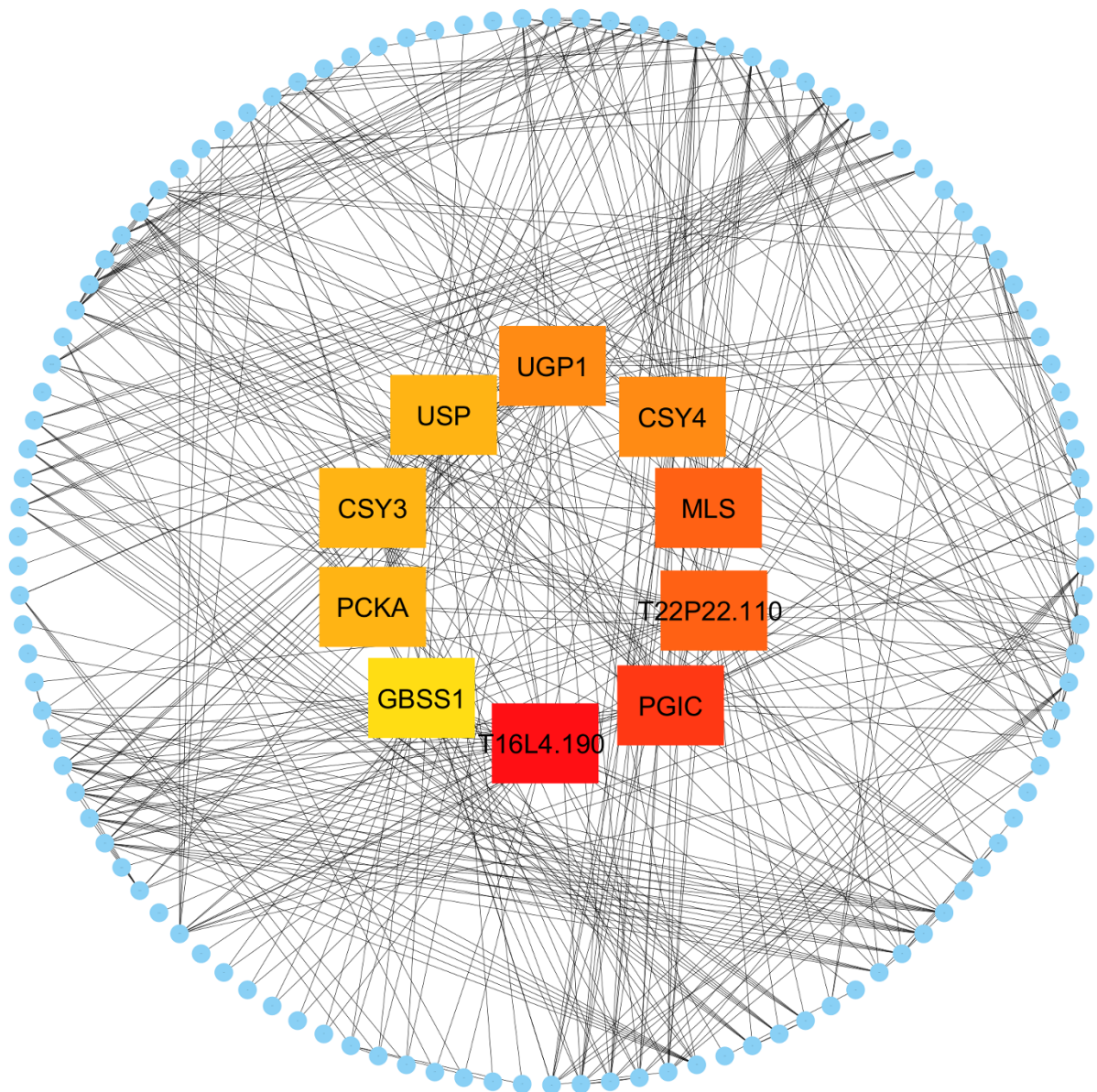

C2

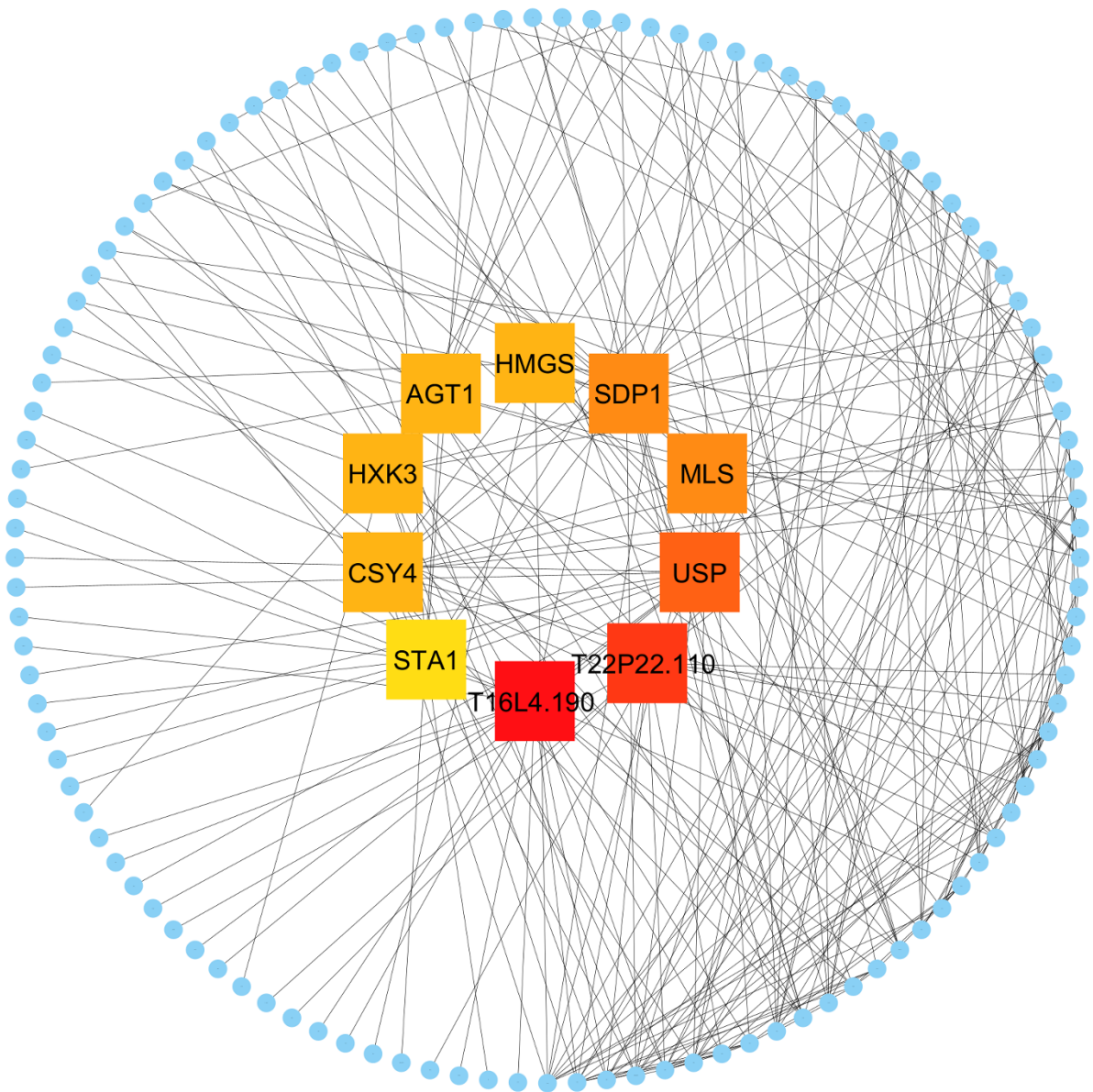

C3

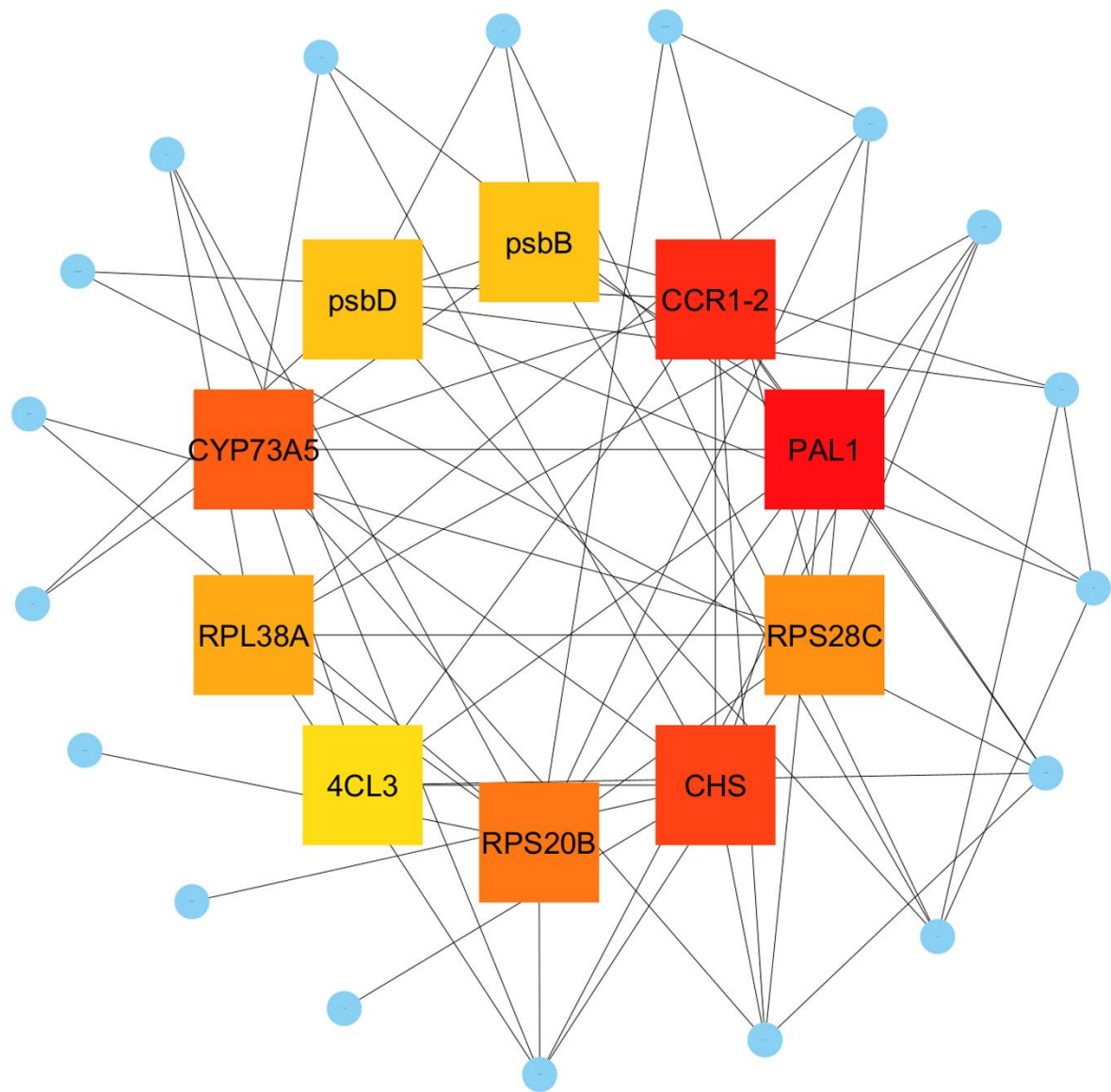

C4

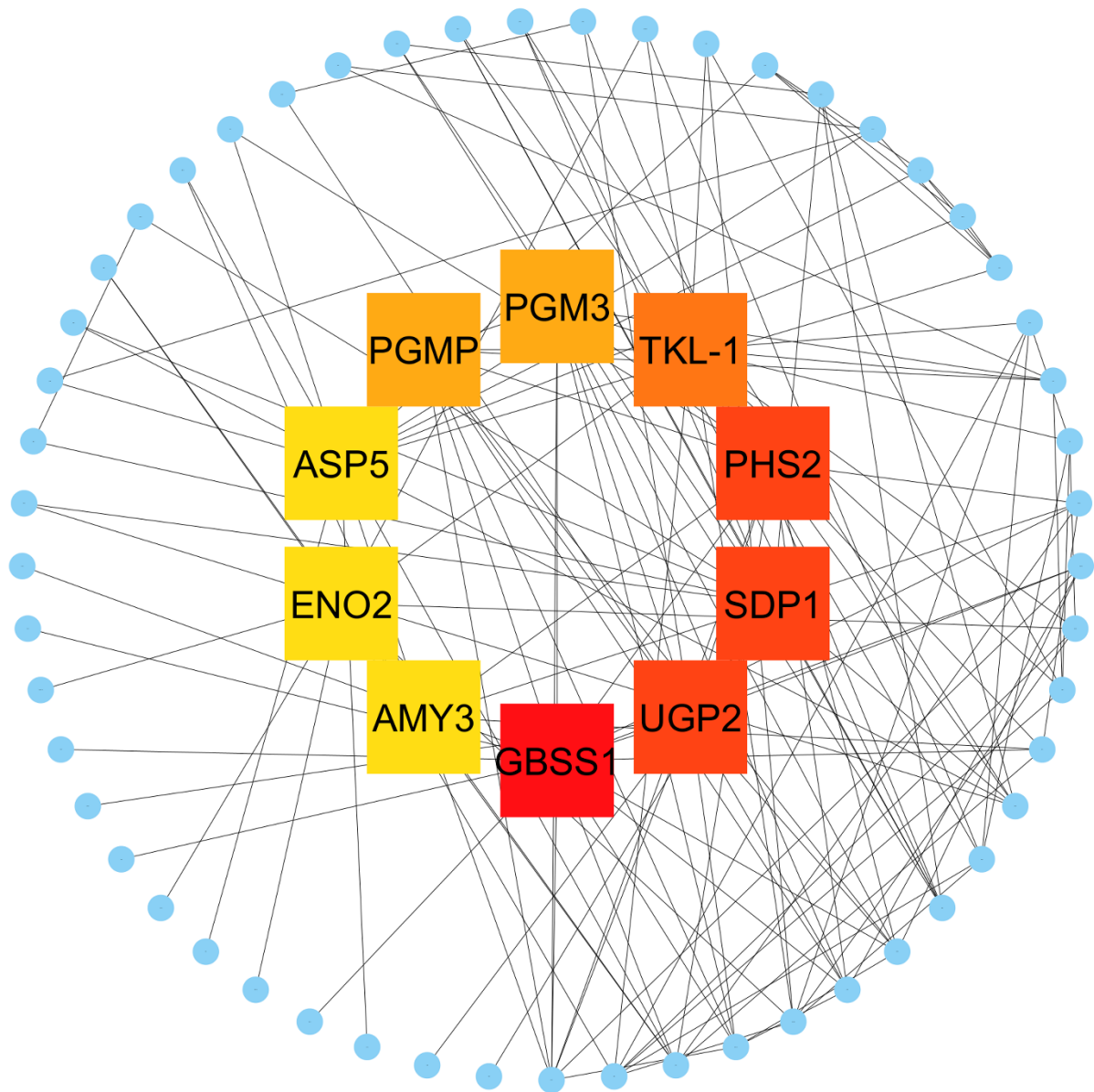

C5

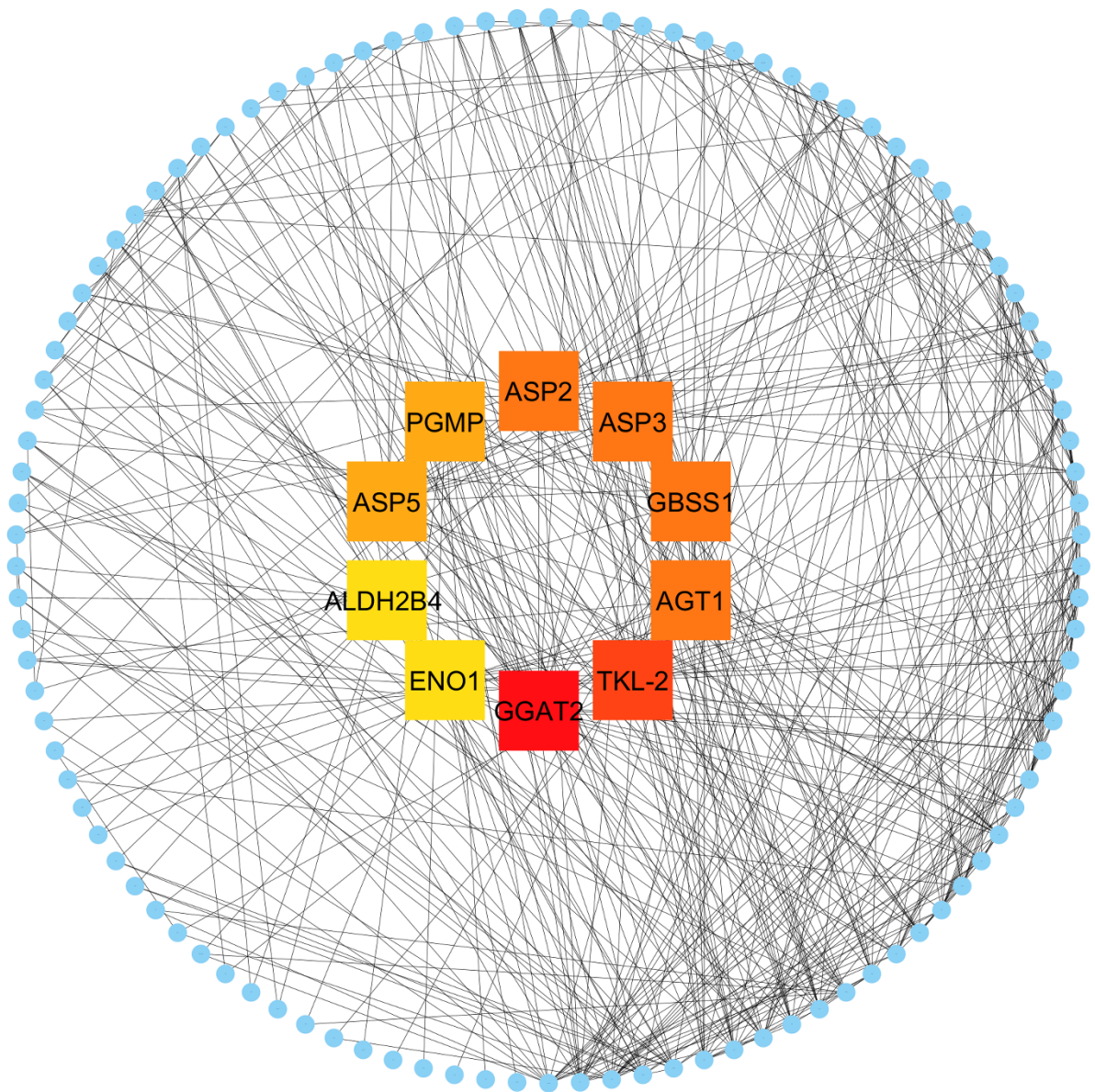

Supplement: Supplementary file 14 — Supplementary Material 14. [file 12870_2025_7102_MOESM14_ESM.pdf]
